# A changepoint approach to automated estimation of soil moisture drydown parameters from time series data

**DOI:** 10.1038/s41598-025-27067-w

**Published:** 2025-12-02

**Authors:** Mengyi Gong, Jessica Davies, Rebecca Killick, Christopher Nemeth, Shangshi Liu, John N. Quinton

**Affiliations:** 1 School of Mathematical Sciences, Lancaster University, Lancaster, UK; 2 Lancaster Environment Centre, Lancaster University, Lancaster, UK; 3 Department of Earth and Environmental Sciences, The University of Manchester, Manchester, UK; 4 Yale School of the Environment & Yale Center for Natural Carbon Capture, Yale University, New Haven, CT USA

**Keywords:** Soil moisture time series, Soil moisture drydown, Changepoint detection, Drydown characteristics, Statistics, Solid Earth sciences

## Abstract

Advances and the growing deployment of *in-situ* soil sensing has the potential to deliver new insights into soil system dynamics. However, it also calls for the development of efficient data analysis methods that can extract interpretable information from continuous data. This study utilises an automated, changepoint-based method for analysing soil moisture time series data. The method is used to autonomously detect wetting events and dynamically estimate parameters describing the drydown characteristics of the soil moisture following the event. Information can then be extracted from the output of the changepoint analysis. This provides an indication of how soils are responding to wetting events, and here we explore if this information corresponds with soil characteristics. In an illustration using soil moisture data from nine different field sites in the United States, different drydown characteristics were observed from the distributions of the estimated parameters. We find that these features can be associated to the climatic regimes and the soil texture of the sites. The potential for identifying changes in soil properties and processes based on shifts in drydown characteristics over time is discussed.

## Introduction

Understanding, monitoring and predicting changes in soil properties has become a major global priority for policymakers and practitioners as soil health has risen up the agenda and they attempt to enable better soil management and enhance the delivery of multiple ecosystem services^1–4^. To date, soil monitoring has focused on the collection of physical, chemical, and biological properties^5^. Many of these measures rely on well-established in-field sampling and laboratory methods. However, the time-consuming and costly nature of field and laboratory work means that these observations often only provide a snapshot of soil conditions over time, leading to a relatively static picture of soil properties. To understand how soil properties and processes may be changing over time in response to pressures and management interventions, it is important that we develop cheaper, more dynamic approaches to soil monitoring.

Improvements and reductions in the cost of *in-situ* continuous soil sensing options opens up new opportunities for a more dynamic picture of soil properties and processes^6,7^. However, as the feasibility of installing multiple *in-situ* soil sensors increases, challenges arise in processing large data volumes and reliably and repeatably extracting meaningful insights from these data.

As our capacity to sense soil properties and processes grows, we must also develop our ability to analyse and interpret soil time series data. The growth in data science and machine learning techniques over the past decades offers many opportunities for developing this capacity. Data science methods are increasingly used in environmental fields, including soil sciences^8^. However, most applications of machine learning have been focused on geospatial analysis of soils^9–13^. This is likely due to the availability of data from soil sampling, soil surveys and remote sensing, which are typically dense in space but sparse in time, and the need to develop and improve mapped products. Whilst spatial modelling is important in understanding soil resources, these products are typically relatively static snapshots of soil properties and conditions. Very few studies have sought to interpret temporal changes in soil properties and processes from time series data^14,15^.

Hence, there is a need to develop approaches that robustly and efficiently extract physically interpretable properties relevant to soil over time, from large time series data, in an autonomous manner. Despite the wide application of machine learning methods, they are often considered as “black box” in nature. Interpretability has been highlighted as a key gap in a recent review^8^. To provide meaningful, actionable information for soil management, data analysis methods that provide insight into biological, chemical, and physical properties and processes of soils are needed.

Here, we begin to address this gap by presenting a data-driven approach to analyse *in-situ* soil moisture time series data, with the aim of generating insights into the dynamics of soil moisture. We focus on soil moisture because high temporal resolution *in-situ* sensing of soil moisture is relatively well-established compared to sensing of other soil variables, such as $\textrm{CO}_{2}$ and soil chemical parameters. How water is stored and moves through soils is affected by multiple soil properties, such as texture, porosity, pore size distribution, surface conditions, and organic matter content. Hence, we expect soil moisture dynamics to vary with soil properties. The dynamics also respond to a multitude of climatic properties, including rainfall patterns and temperature. Despite the relative maturity of soil moisture sensing, few studies have aimed to autonomously extract insights into how it responds in different soil and climatic settings from *in-situ* time series. Past studies have used *in-situ* or remotely sensed soil moisture data to develop data-driven models that reproduce soil moisture dynamics^16^, or provide insights as to when to schedule irrigation^17^. Here we explore if a data-driven method can provide insights into the dynamics of soil moisture from time series data across a suite of sites with varying soil types and climatic regimes.

In what follows, we apply a new changepoint-based method for modelling soil moisture dynamics to soil moisture time series data, where the peaks in the soil moisture time series are autonomously detected, and parameters of drydown curves^18^, which are the decreasing segments following the peaks, are estimated on an event-by-event basis, such that changes in these parameters can be observed over time. This approach is then used to estimate soil moisture travel times down the soil profile, by simultaneously analysing and integrating data from sensors at multiple depths in the profile. We apply these approaches to nine field sites with a variety of soil types and climatic regimes across the US, from the United States National Ecological Observation Network (NEON, https://data.neonscience.org/). These sites provide a variety of conditions and a set of long-term high-frequency soil moisture time series derived from similar equipment and monitoring set-ups. We hypothesise that drydown parameters and travel time distributions at different sites will vary depending on climate and soil compositions. This analysis is intended to provide a proof-of-concept that insights relevant to soils and climate regime can be autonomously extracted from dynamic soil moisture data from sensors. We posit that this method could be built upon with further studies examining sites where there are known changes in soil conditions to test if this is a viable approach to detecting soil property changes over time.

## Data and methods

### Soil moisture data collection

Continuous soil moisture (in volumetric water content) time series were obtained from NEON using soil capacitance probes (Sentek TriSCAN). In particular, measurements are made using soil moisture sensors in vertical profiles consisting of up to eight depths in five soil plots instrumented with soil capacitance probes in each field site. The time series data are presented as 1-minute and 30-minute averages. From the initial installation of the sensors up to early to mid 2019 (depending on field sites), the manufacturer’s default calibration was used (which is $Y= a X^{b} + c$, where $a=0.19570$, $b=0.40400$, $c=0.02852$^19^). After that, a soil-specific calibration was used, which was then reverted back to the manufacturer’s method between late 2021 to early 2022. This resulted in visible discontinuities in the time series data. In addition, soil moisture sensors cannot accurately measure frozen water, so the measurements during the period when the soil is frozen are flagged^20^, resulting in regular winter gaps in some time series. This prohibits the analysis of soil moisture measurements from many field sites. As a consequence, nine field sites where there is at least one time series that displays no major gaps over a period of at least 12 months, or with only a missing gap during the frozen period, were selected (see Table 1). A summary of the climate and soil properties, including mean annual precipitation (MAP), mean annual temperature (MAT), soil types, soil composition and vegetation types, of the nine field sites was given in Tables 2 and 3. Locations of the field sites are shown in the maps of soil texture and mean annual temperature in Figure S2 in section 3 of the supplemental document.

The nine field sites cover a wide range of soil, climate and ecosystem types. Mean annual precipitation ranged from 288 mm at ONAQ, Utah, to 1328 mm at TALL, Alabama, and mean annual temperatures ranged from 4.3 $^{\circ }$C for UNDE, Indiana, to 20.9 $^{\circ }$C for OSBS, Florida. Soil types cover six out of twelve USDA Soil Taxonomy soil orders^21^. High sand content is found in OSBS, TALL, Florida (97%), TALL, Alabama (90.7%), SRER, Arizona (78.8%) and CPER, Colorado (71.5%). High silt content is found in GUAN, Puerto Rico and ORNL, Tennessee, with a percentage of over 50%. Two field sites with relatively high clay content are GUAN, Puerto Rico (34.9%) and SCBI, Virginia (32.1%). The vegetation types (IGBP classification) include Grasslands, Open Shrublands, Deciduous Broadleaf Forests, Evergreen Broadleaf Forests, Evergreen Needleleaf Forests and Mixed Forests.

Time series data of the nine field sites are accessed via https://data.neonscience.org/data-products/DP1.00094.001. In particular, the 30-minute averages from the shallowest depth that has the best quality among all time series from the five soil plots of each field site were selected. The selected time series have a length of 12 months, unless the site experienced some form of winter freeze, in which case, a minimum length of 10 months is required. The 30-minute time series were first down-sampled to hourly time series before the gaps due to missing observations were filled via linear interpolation. The majority of the missing gaps in the selected time series are short ($\le 12$ hours). The maximum missing gap of 3.9 days is found in site CPER. The resulting time series have a length of around 8,000 time points.

**Table 1:** Summary of the selected soil moisture time series: study periods and maximum missing gaps.

Site	Location	Study period	Max gap
SRER	Arizona	June 2018 - May 2019	$< 1$ day
TALL	Alabama	Feb 2018 - Jan 2019	3.8 days
OSBS	Florida	Jan 2018 - Dec 2018	1.1 days
UNDE	Michigan	March 2020 - Feb 2021	$< 1$ day
CPER	Colorado	mid March 2021 - Dec 2021	3.9 days
SCBI	Virginia	March 2021 - Dec 2021	$< 1$ day
ONAQ	Utah	Feb 2021 - Jan 2022	$< 1$ day
GUAN	Puerto Rico	Jan 2021 - Dec 2021	3.1 days
ORNL	Tennessee	March 2020 - Feb 2021	$< 1$ day

**Table 2:** Summary of climate regimes and soil types of nine selected NEON sites (part I).

Site	Climate	Soil type	Soil content	Vegetation
CPER - Central Plains Experimental Range, Pawnee National Grasslands, Colorado	Bsk (Steppe: warm winter); 1600 m above sea level; MAT 8.6 $^{\circ }$C; MAP 344 mm.	Order: Mollisol; Family: Fine - loamy - mixed - superactive - mesic Aridic Argiustolls	Sand total: 71.5%; Silt total: 16.6%; Clay total: 11.9%	Grasslands; moderately grazed shortgrass steppe; dominant plants include blue grama, buffalograss, and plains prickly-pear cactus
GUAN - The Guanica Dry Forest Reserve, southern coast of Puerto Rico	Am (Tropical monsoon); MAT 23.0 $^{\circ }$C; MAP 840 mm	Order: Aridisol; Family: Coarse - loamy - carbonatic - isohyperthermic Typic Haplocalcids	Sand total: 7.1%; Silt total: 57.4%; Clay total: 34.9%	Evergreen Broadleaf Forest; plant species include cacti, grasses, and shrubs and the forest has of areas with semi-evergreen, deciduous and scrub trees
ONAQ - Onaqui, Southwest of Salt Lake City, Utah	Dfb (Warm Summer Continental: significant precipitation in all seasons); MAT 9.0 $^{\circ }$C; MAP 288 mm	Order: Aridisol; Family: Fine - loamy - mixed - superactive - mesic Xeric Haplocalcids	Sand total: 59.5%; Silt total: 28.3%; Clay total: 12.2%	Open Shrublands; eastern half dominated by Big Sagebrush; along the base of Onaqui Mountains the vegetation transitions into Utah Juniper and Pinyon Pine woodland
ORNL - Oak Ridge National Laboratory, Cumberland Plateau, Tennessee	Cfa (Humid Subtropical: mild with no dry season, hot summer); MAT 14.4 $^{\circ }$C; MAP 1340 mm	Order: Ultisol; Family: Fine - kaolinitic - thermic Typic Paleudults	Sand total: 29.2%; Silt total: 56.3%; Clay total: 14.5%	Deciduous Broadleaf Forests; world’s largest hardwood-forested plateau; canopy dominated by oaks and hickories
OSBS - Ordway-Swisher Biological Station, Florida	Cfa (Humid Subtropical: mild with no dry season, hot summer); MAT 20.9 $^{\circ }$C; MAP 1302 mm	Order: Entisol; Family: Hyperthermic - uncoated Typic Quartzipsamments	Sand total: 97%; Silt total: 2%; Clay total: 1%	Evergreen Needleleaf Forests; dominated by pine and turkey oak vegetation with a grass and forb groundcover

Source: https://www.neonscience.org/field-sites/explore-field-sites.

**Table 3:** Summary of climate regimes and soil types of nine selected NEON sites (part II).

Site	Climate	Soil type	Soil content	Vegetation
SCBI - Smithsonian Conservation Biology Institute, the Blue Ridge Mountains, Virginia	Cfa (Humid Subtropical: mild with no dry season, hot summer); MAT 11.6 $^{\circ }$C, MAP 1126 mm	Order: Alfisol; Family: Loamy - skeletal - mixed - active - mesic Ultic Hapludalfs	Sand total: 16.4%; Silt total: 51.5%; Clay total: 32.1%	Deciduous Broadleaf Forests; the mature forests are primarily oak, hickory, ash, and tulip poplar; the young forests are primarily white ash, black locust, and dogwood
SRER - Santa Rita Experimental Range, Arizona	Bsk (Steppe: warm winter); MAT 19.3 $^{\circ }$C, MAP 346 mm	Order: Entisol; Family: Coarse - loamy - mixed - calcareous - thermic Typic Torrifluvents	Sand total: 78.8%; Silt total: 16.2%; Clay total: 5%	Open Shrublands; dominated by drought-resistant, thorny species, including a mix of short trees, shrubs, cacti and other succulents, perennial grasses, and annual forbs.
TALL - Tallagada National Forest, Alabama	Cfa (Humid Subtropical: mild with no dry season, hot summer); MAT 17.2 $^{\circ }$C, MAP 1328 mm	Order: Ultisol; Family: Fine - loamy - siliceous - subactive - thermic Typic Hapludults	Sand total: 90.7%; Silt total: 6.3%; Clay total: 3%	Evergreen Needleleaf Forests; dominated by conifers, with some areas of intermixed conifers, hardwoods, bottomland hardwoods, and wetlands.
UNDE - University of Notre Dame Environmental Research Center, Michigan	Dfb (Warm Summer Continental: significant precipitation in all seasons ); MAT 4.3 $^{\circ }$C, MAP 802 mm	Order: Spodosol; Family: Coarse - loamy - mixed - superactive - frigid Argic Fragiaquods	Sand total: 62.3%; Silt total: 32%; Clay total: 5.7%	Mixed Forests; primarily second-growth Northern mesic forest; dominant species of the area are red and sugar maple, aspen, and paper birch.

Source: https://www.neonscience.org/field-sites/explore-field-sites.

### The changepoint approach to modeling soil moisture decay

**Fig. 1:**
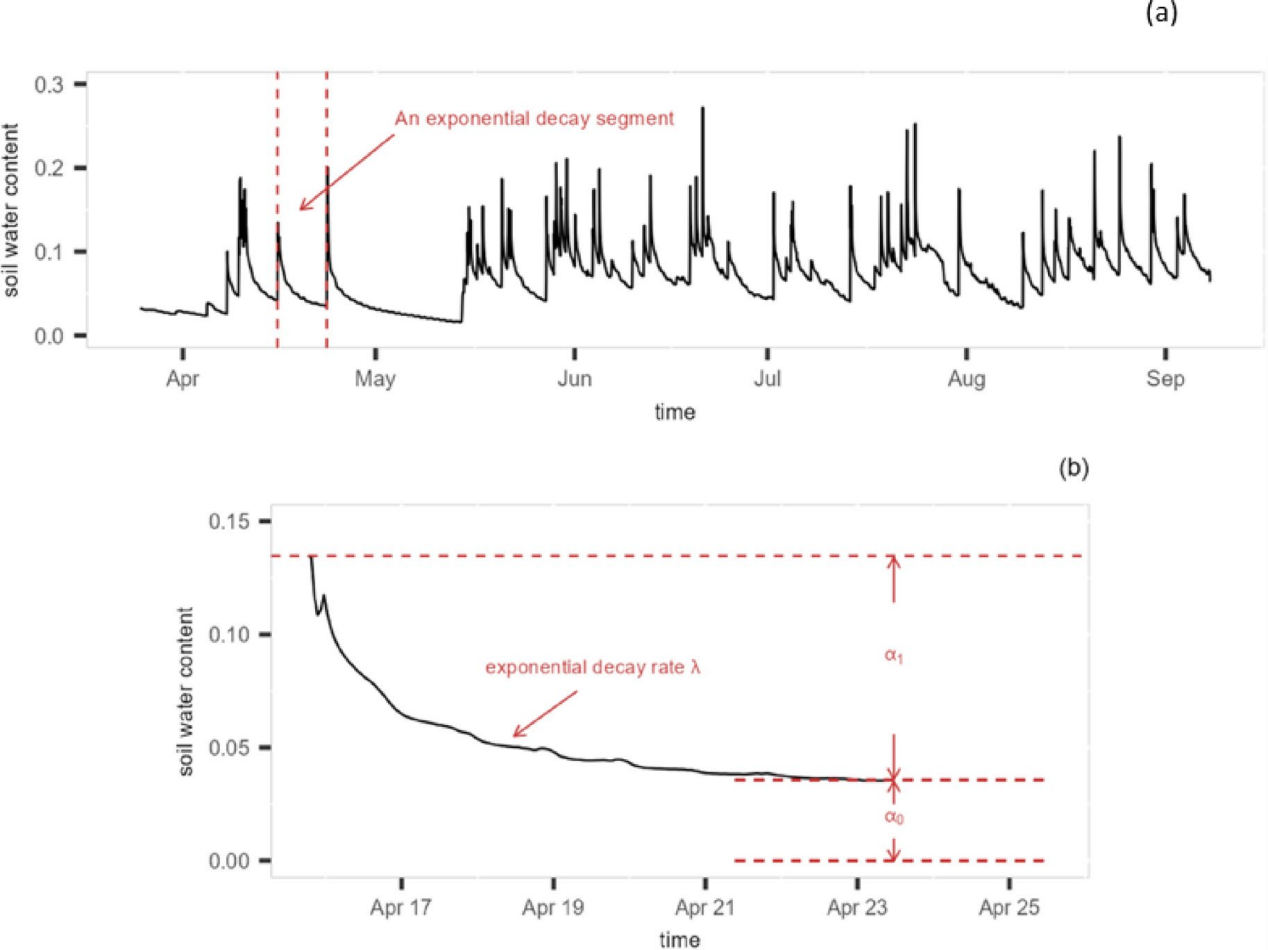
(**a**) An example of soil moisture time series from site OSBS. The segment between two red dashed lines represents an exponential decay process. (**b**) Illustration of the exponential decay model parameters on a close-up of a decreasing segment.

A common structure displayed in the soil moisture time series is a gradual decrease process following a peak in soil water content as a result of, e.g., precipitation. The decrease of soil water content is a result of drainage, runoff and evapotranspiration (ET). The soil water loss process typically begins with a drainage and runoff dominated phase when soil moisture level is high, followed by ET-dominated phases at moderate and low soil moisture levels^22^. The decrease continues until the next weather event disrupts the process and causes the soil moisture to rise again, reaching the next peak. Figure 1a presents an example of the soil moisture time series from field site OSBS, and Fig. 1b shows a close-up of a decreasing segment.

The drainage dominated phase usually happens during and shortly after the weather event, whereas the ET dominated phase can last much longer^18,23^. In fact the drainage dominated phase may not be observable in low frequency time series, e.g., satellite data that are obtained every few days. Therefore, historically, analyses in literature have been focused on the ET dominated phase^18,24,25^. Typically, the decreasing segment is modelled using a soil moisture drydown model^18^, which can be written as

1 $$\begin{aligned} \theta (t) = \Delta \theta \exp \left( -\frac{t}{\omega } \right) + \theta _{f} \, . \end{aligned}$$

Here, $\theta (t)$ represents the soil water content at time *t*, $\Delta \theta$ is the increase of soil water content caused by the weather event, $\theta _{f}$ is the estimated lower bound of the soil moisture observations, and $\omega$ is the “temporal e-folding decay”, which describes the time it takes for the soil water content to drop to $\frac{1}{e}$ of its initial level^18^. Prior studies have typically focused on the spatial and temporal variations in the estimated drydown parameter $\omega$^25–28^, and the results are often presented as (seasonal) maps of the estimated e-folding time scale.

The first step in drydown modelling is the identification of the drydown curves from a soil moisture time series. This involves manual processing of the soil moisture time series, along with supporting data, such as the precipitation time series. This may encounter problems when long-term high frequency data are concerned. First of all, the large number of observations means that manual processing can be exhaustive. The finer details captured by the high frequency time series, e.g., diurnal cycles, suggests that the usual identification criteria, such as a continuous period of negative increments of soil water content at each time step, need to be refined. Secondly, despite the wide availability of precipitation data products, there is sometimes a mismatch between the precipitation time series and the soil moisture time series, where soil moisture peaks without a significant rainfall, or vice versa.

To overcome these challenges, we applied a changepoint based method^29^ to analyse the soil moisture time series and extract meaningful information from the data. The method was designed to automate the processing of long soil moisture time series with minimal requirement on external data or manual power. In a nutshell, consider the sudden increase of soil water content as an abrupt “change” to the soil moisture decreasing process that would have continued without disturbance. Define the time point before the peak as a “changepoint” $\tau _{i}$, then the segment between the current peak and the next peak, i.e., the segment between two adjacent changepoints $\tau _{i}$ and $\tau _{i+1}$, approximately represents the drydown process. Here we use “approximately” to indicate the difficulty in identifying the precise location of the starting point of the drydown curve. This is due to the relatively small number of observations during the wetting phase. However, the impact of these observations is usually small since the drying process often takes much longer. See section 2 of the supplemental document for some discussion, with illustration in Figure S1. The statistical model for such a decreasing segment can be written as

2 $$\begin{aligned} \theta _{t}&= \alpha _{0i} + \alpha _{1i} \, \lambda _{i}^{(t-\tau _{i})} + \epsilon _{t} \\&= \alpha _{0i} + \alpha _{1i} \, \exp (-\exp (\gamma _{i}))^{(t-\tau _{i})} + \epsilon _{t} \nonumber \\ \epsilon _{t}&\sim \mathcal {N}(0, \sigma ^{2}) \nonumber \end{aligned}$$

where $\theta _{t}$ is soil water content recorded by the soil sensors at time *t*, $\alpha _{0i} > 0$ is the lower bound (or the asymptotic level) of the soil water content of the *i*-th segment; $\alpha _{0i} + \alpha _{1i}$ (with $\alpha _{1i} > 0$) is the soil water content at the peak, $\lambda _{i}$ is the rate of exponential decay with $\lambda _{i} \in (0, 1)$, and $\exp (-\exp (\gamma _{i}))$ is a re-parameterisation of the decay rate with $\gamma _{i} \in \mathbb {R}$. The decay rate can be converted to the e-folding decay via $\omega = -\frac{1}{\log (\lambda )}$. Model (2) is essentially the re-parameterised drydown model (1) with additional random component $\epsilon _{t}$, where $\epsilon _{t}$ is used to reflect the uncertainty or noise in the data. An illustration of the model parameters is given in Fig. 1b. To enable the model to capture potential temporal patterns in the data, all parameters in model (2) are given the flexibility to change from segment to segment. As the soil moisture decay process is related to not only soil properties, but also climate, vegetation, human activities, etc., using segment specific parameters can better characterise the temporal dynamics in the system.

Estimation of the changepoints and the model parameters are carried out simultaneously via minimising a penalised overall cost function using the penalised exact linear time method^30^. It is important to note that precipitation data are not essential to the changepoint method, and are not used in the analyses presented here. However, they could be used to guide the changepoint detection process via penalty learning^29,31^. The penalty parameter is applied to avoid over-fitting i.e., estimating too many changepoints. For this purpose, data on the timing or frequency of precipitation events can provide useful guidance. The changepoint method can be implemented using the R code developed by the authors. A brief introduction of the method is given in section 1 of the supplemental document.

Finally, it should be acknowledged that with high-resolution time series data such as that used in this study, the soil moisture data following an event-driven peak include both drainage- and ET-dominated phases of drydown. Various methods have been proposed to estimate the drydown rate (referred to as the “short term memory” in literature) during the drainage-dominated phase^23,32^. Their estimation methods rely on pre-defined arbitrary thresholds or sampling intervals to isolate the drainage dominated period from the ET-dominated period. For high-frequency data, however, a more accurate representation of the transition between the two phases may be required to actually avoid over or underestimation. It is statistically possible to find such a transition point through another round of changepoint detection with the aim of identifying the timing when the drydown rate experiences a significant change (see section 4 for an example). However, to verify any method that aims to locate the transition point would require specific lab experiments or field studies for evidence. Therefore, the separation of the drainage phases from the evapotranspiration phases was not considered in this study. Instead, we chose to represent both phases with one curve. This is the simplest approach in the absences of further information and we consider it valid for our purposes as both phases are influenced by climatic and soil property drivers.

### Analysing the output of the changepoint model

The output of the changepoint analysis includes the estimated changepoints, which reflect the timing of the peaks in the soil moisture time series, and the estimated exponential decay model parameters for each segment, $\alpha _{0i}$, $\alpha _{1i}$ and $\gamma _{i}$, for $i = 1, \cdots , k$, if *k* segments are identified by the algorithm. These estimations enable us to carry out various post-analyses to investigate the characteristics of soil moisture drydown of a particular field site, and make comparisons between field sites. Apart from the widely used summary statistics, such as mean, median and variance, we can also visualise the result as time series to explore temporal patterns, and investigate the time lags between peaks identified in soil moisture time series from different depths along the soil profile. Some details on the post-analyses performed in this study are given below.

#### Single-depth analysis

**Fig. 2:**
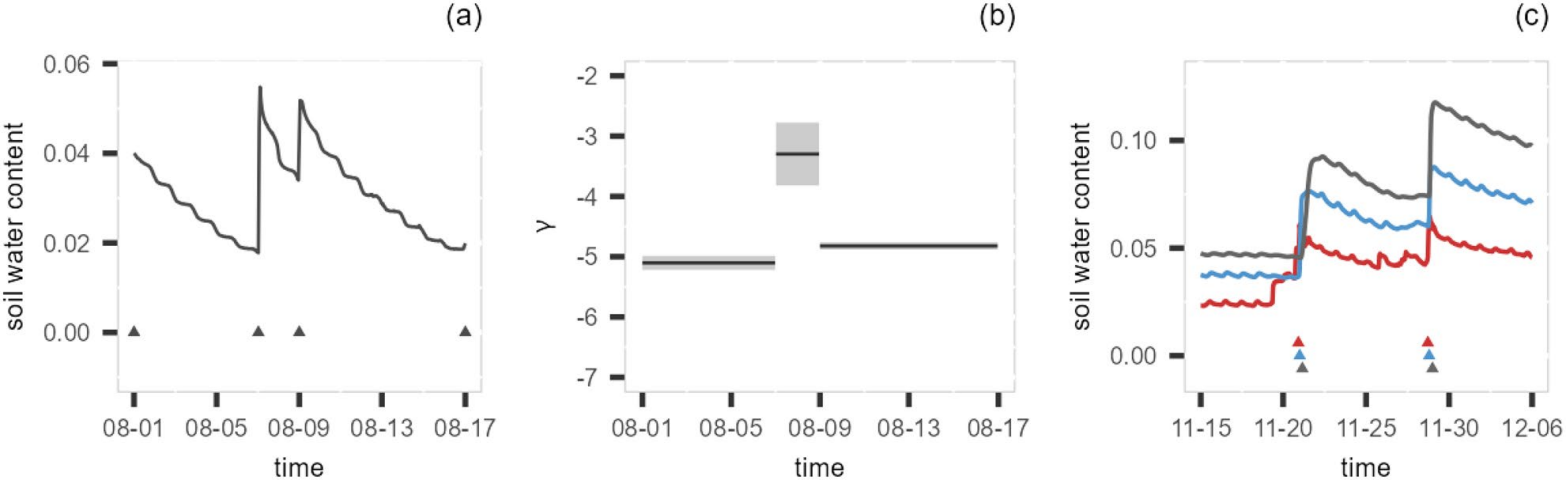
Illustrations of, (**a**) the soil moisture time series with the detected changepoints (black triangles), (**b**) the piecewise constant time series plot of the estimated decay parameter $\gamma$ with confidence intervals (grey shaded rectangles), (**c**) the identified matching changpoints from the same drydown event at top (red), middle (blue) and the bottom (grey) depths.

The estimated parameters can be visualised as piecewise constant time series to explore the potential temporal patterns. To do this, we can use a horizontal line that stretches over the period of the corresponding segment to represent the estimated parameter value for a particular drydown event. Uncertainties of the estimated parameters (i.e., the standard errors) can be visualised as shaded areas around the horizontal lines. Figure 2 presents an illustration of visualising the changepoint model output, where panel (a) shows the data and the estimated changepoints, and panel (b) shows the piecewise constant time series plot of the decay parameter $\gamma$. For comparisons between sites, histograms of the estimated parameters can be used, along with the conventional summary statistics. To investigate the association between soil moisture drydown characteristics, climate types and soil compositions across sites in this study, the quantiles of the estimated parameters are correlated to the MAP, MAT, and soil clay, sand, silt contents of different field sites. To explore seasonality in the data, one could correlate the standard deviation of the estimated parameters to a seasonality index, which measures the intra-annual rainfall variability^33^. However, this requires the long-term monthly summary of precipitation, which is not available in this case.

Considering the nature of the nonlinear least squares method used for estimating the drydown model parameters, segments with boundary solutions or high uncertainty in parameter estimation are excluded from the analysis. The discarded segments often do not display a clear exponential decay pattern, and hence the poor parameter estimation. This can happen during the winter period when the soil is close to saturation, or when the wetting segment is relatively long and hence is separated from the remaining drying segment.

#### Multi-depth analysis

Applying the changepoint detection method to soil moisture time series recorded at different depths along the soil profile enables us to explore the time lags between the estimated changepoints at different depths. Such time lags, referred to as “travel time” hereafter, between the peaks in a shallower layer and a deeper layer can provide some insight into the infiltration of the water into the soil layers. To estimate the travel times of the peaks, we first identify all changepoints in a shallower layer that have a matching changepoint in a deeper layer, then we calculate the delays between the matching changepoints and keep the ones that are below certain threshold. The threshold is used to distinguish the peaks of the current drying event from the peaks of the adjacent drying event. In this case, two changepoints were matched if the travel time was less than 24 hours between the sensors in the top and middle layers (10cm apart) and less than 72 hours between the sensors in the top and bottom layers (20cm apart). These maximum delay thresholds were chosen to encompass the typical ranges of infiltration rates for soils and acknowledge that permeability generally reduces with depth^34–37^.

This multi-depth analysis idea is illustrated in panel (c) of Fig. 2, where it presents the soil moisture time series measured at three different depths (red for top, blue for middle and grey for bottom layers), with a 10 cm gap between each layer, along with the changepoints (coloured triangles) that were identified to have at least one match in a deeper layer. The time distance between the matched triangles corresponds to the travel time. Note that not all peaks spotted in shallower layers have matching peaks in the deeper layers, as the water may not infiltrate far enough to reach the lower level.

## Results

**Fig. 3:**
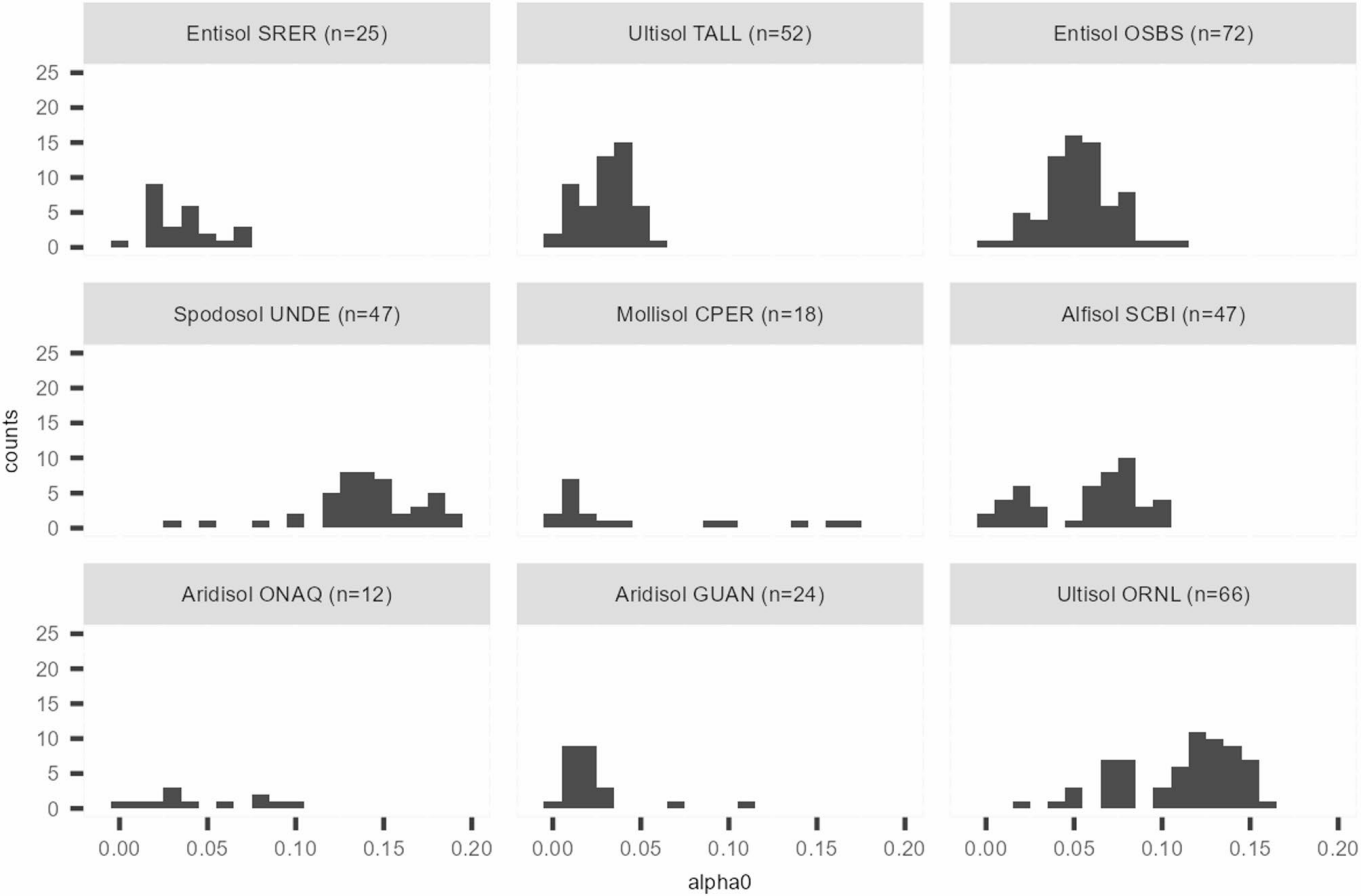
Histograms of estimated asymptotic parameter $\alpha _{0}$ from nine NEON field sites. The letter *n* stands for the number of drydown events consisting of the histogram.

The changepoint method was first applied to the soil moisture time series from nine NEON field sites to extract the distributions of the parameters of the exponential decay model (2). The two parameters of interest in the exponential decay model are the asymptotic soil moisture level $\alpha _{0}$ and the decay rate $\lambda$. The histograms of the estimated $\alpha _{0}$ and $\lambda$ are presented in Figs. 3 and 5, respectively. The correlations between quantiles (i.e., the median, 1st and 3rd quartiles) of the estimated $\alpha _{0}$ and $\lambda$ and MAT, MAP, proportions of sand, silt and clay of the soil are presented in Table 4, where the pairs of variables with significant positive or negative correlations are highlighted. These results are discussed by parameter in the sections that follow. We also present the summary statistics of the estimated parameters for all sites in Table S1 and the time series plots of soil moisture, changepoints and estimated parameters for all sites in Figures S3 to S11 in section 3 of the supplemental document.

In a second analysis, the changepoint method was applied to time series from the top three soil profile layers at various soil plots within NEON field sites SRER, OSBS and TALL to explore the distribution of the travel times of the peaks across the layers. The remaining six field sites do not have enough data of good quality for the top three layers. Hence, they are excluded from this analysis. The travel times (in hour/cm) provide a unique perspective to explore the speed of infiltration in different field sites. Histograms of the delays are given in Fig. 6. Plots of the soil moisture time series from three layers for all sites, along with the matching changepoints are presented in Figures S12 to S18 in section 4 of the supplemental document.

Note that since the soil in these NEON field sites were undisturbed during the monitoring period, the aim of the analysis was to establish some metrics that can reflect the natural variations of the parameters. Here obtaining different values of $\alpha _{0}$ and $\lambda$ from the changepoint analysis does not mean the soil was changing rapidly during the monitoring period. Soil moisture decay is a complex process. Variation in antecedent conditions or external conditions can all affect the decay rates and the asymptotes. There is also the impact from the sensor noise. Thereby, the variation in the estimated parameters are meant to capture the uncertainty resulting from these complexities. Sometimes, the variation corresponds to a seasonal pattern, whereas other times they may not be easily interpreted.

**Table 4:** Correlations between the 1st quartile, median, 3rd quartile of the estimated asymptotic parameter $\alpha _{0}$, decay rate $\lambda$ and climate variables, soil compositions.

	$\hat{\alpha }_{0}$			$\hat{\lambda }$		
	1st qt	median	3rd qt	1st qt	median	3rd qt
MAT (deg)	-0.6603*	-0.6996*	-0.8016***	0.1556	0.1698	0.0713
MAP (mm)	0.2642	0.3336	0.1889	-0.6267*	-0.6423*	**-0.5486**
sand total	-0.0704	-0.1770	-0.0985	-0.2377	-0.3324	-0.3423
silt total	0.2542	0.3517	0.2746	0.1722	0.2522	0.2704
clay total	-0.2558	-0.1452	-0.2173	0.3249	0.4346	0.4290

Significance level from a Pearson correlation test is indicated using stars where *** ($\le 0.01$), ** ($\le 0.05$), * ($\le 0.1$).

### The estimated asymptotic parameter $\alpha _{0}$

The nine sites display different distributions for the asymptotic soil water content $\alpha _{0}$ (Fig. 3). The distributions for CPER and GUAN appear to be right (or positively) skewed; whereas UNDE shows a left (or negatively) skewed distribution. The distributions of TALL and OSBS appear to be more symmetrical and concentrated; whereas those of SCBI and ORNL show signs of a bimodal pattern. The differences can be associated to the climate and soil compositions.

A relationship between $\alpha _0$ and the climate variables (MAT, MAP) can be identified based on the result in Table 4. Across the nine sites there is a strong negative correlation between the median and quartiles of $\alpha _0$ and MAT, while MAP is weakly correlated to the median of $\alpha _0$. Two Aridisols, ONAQ and GUAN, have maximum $\alpha _0$ values below 0.1, indicating that these soils are frequently dry in line with the likely high evaporation and transpiration losses experienced at GUAN and the low MAP at ONAQ (288 mm). CPER (Mollisol) has the lowest median and minimum $\alpha _0$ values, consistent with dry hot summers experienced there, and a high maximum, consistent with winter storms and snow melt. The climatic effect is also evident when comparing the two Entisols (SRER and OSBS). SRER has relatively few drydown events and a maximum $\alpha _0$ of 0.07, again in line with its semi-arid climate and 342 mm MAP. In contrast, OSBS, which has a MAP of 1302 mm, has more events and a distribution which is closer to normal with a maximum $\alpha _0$ of 0.13. The distribution for UNDE (Spodosol) is distinctive amongst the nine sites as this location is climatically different to the others. Being in a cooler climate (MAT = $4.3^{\circ }\textrm{C}$), UNDE has higher $\alpha _{0}$ (above 0.1) for the majority of the year and lower asymptotes only in summer months.

**Fig. 4:**
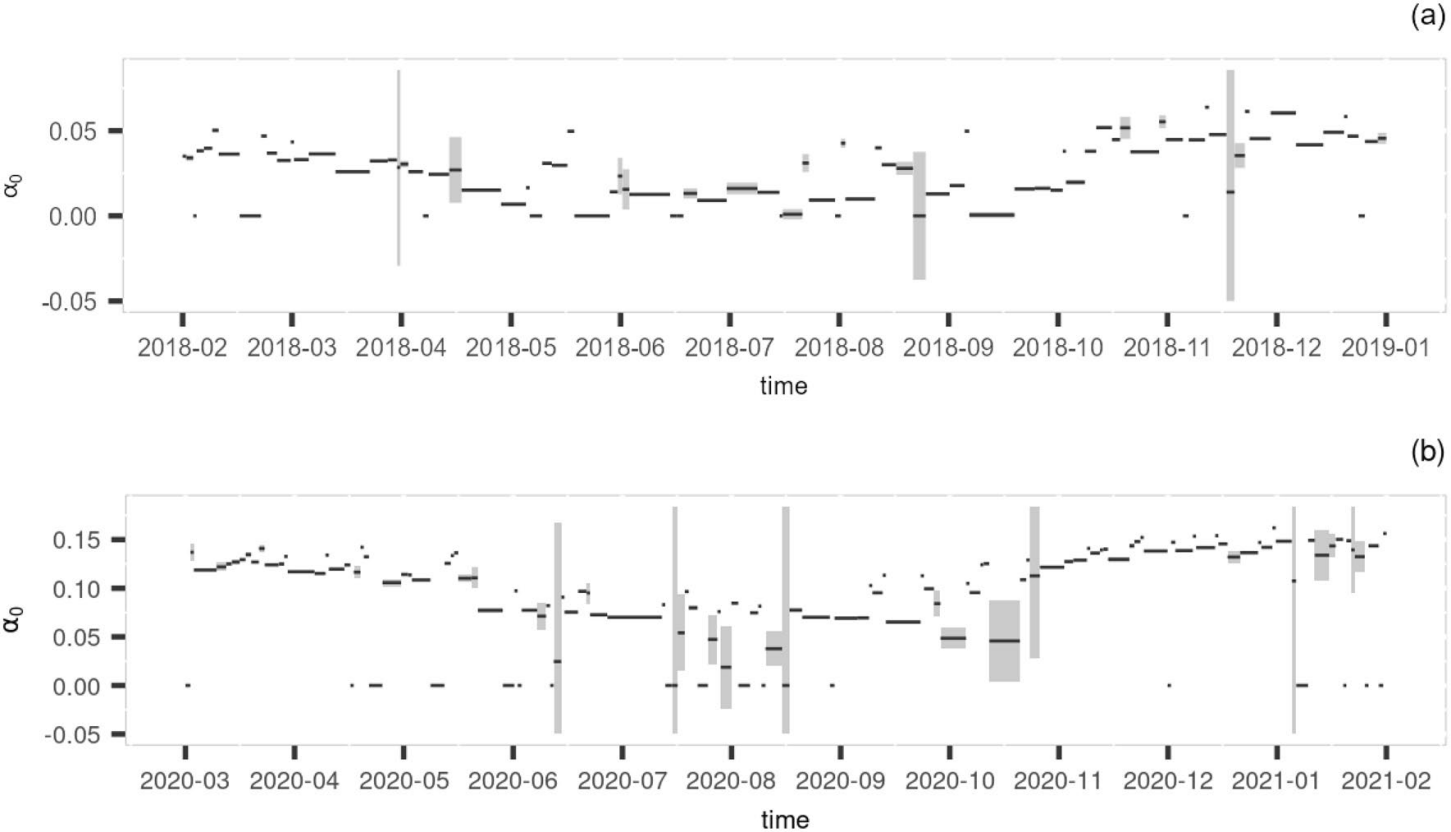
The estimated asymptotic parameter $\alpha _{0}$ plotted over time along with the confidence intervals for site TALL (**a**) and ORNL (**b**).

Potential seasonality can be seen from the time series plot of the parameter $\alpha _0$ for TALL and ORNL (Fig. 4). Whilst the histogram of $\alpha _0$ in TALL is unimodal, the histogram of $\alpha _0$ in ORNL shows sign of a bimodal distribution, which is potentially related to the different asymptotes in summer and winter. The histogram of site SCBI also displays a bimodal feature. However, the seasonal pattern is less evident in SCBI (see Figure S8 in the supplemental document). A longer time series covering several years may be required to draw conclusions on the seasonal variation of the parameters.

Figure 3 illustrates that soil textural properties also influence the distribution of $\alpha _0$. This is best observed in the two Ultisols, TALL and ORNL, which exhibit different distributions of $\alpha _0$. TALL has $\alpha _0$ values that are mostly lower than values found at ORNL. This is despite TALL and ORNL having similar annual rainfall totals of 1328 and 1340 mm respectively. We hypothesise that the lower $\alpha _0$ for TALL is due to its sandy texture, presumably with higher permeability and lower water holding capacity, compared to the silty textured soil at ORNL, which we expect to have lower permeability, but higher water holding capacity. Textural differences can also be observed between the two Aridisols at ONAQ and GUAN. ONAQ has a fine loamy texture, whereas GUAN has a coarse loamy texture. This results in a higher $\alpha _0$ for GUAN, although there are fewer rainfall events occurring in these arid climates, making data interpretation difficult. Across the nine sites, only weak correlations were found between silt content and the median value of $\alpha _0$ (see Table 4).

### The estimated decay parameter $\lambda$

**Fig. 5:**
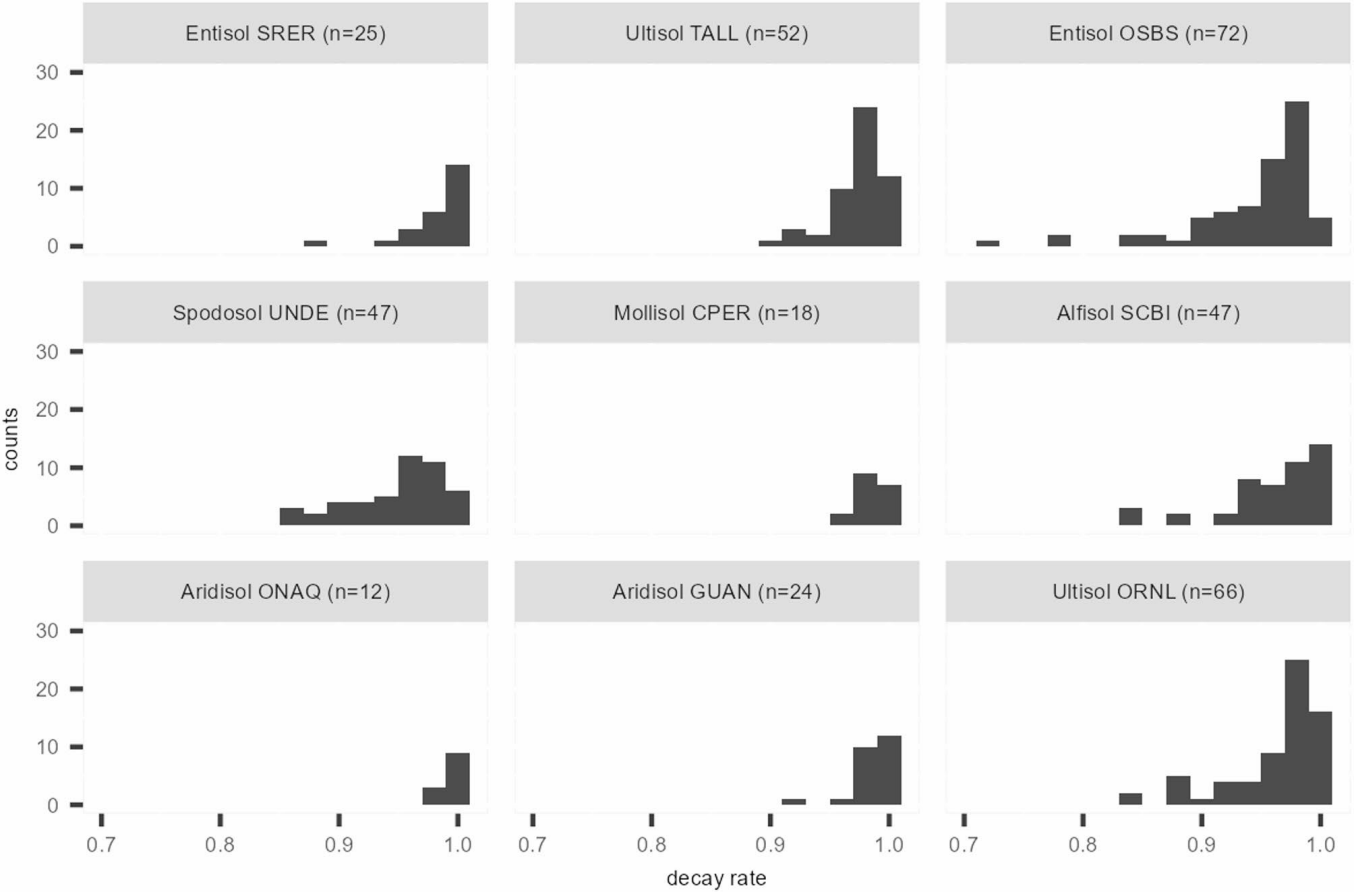
Histograms of estimated decay rate $\lambda$ from nine NEON field sites. The letter *n* stands for the number of drydown events consisting of the histogram. A lower value for $\lambda$ indicates a faster decrease in soil water content.

Figure 5 shows that the Entisol (OSBS) has the widest range of $\lambda$ values, possibly due to the combination of sandy (sand content = 97%), well-drained soil with rainfall that is well distributed throughout the year. In contrast, the Aridisols (ONAQ and GUAN) and the Mollisol (CPER) have slower rates of drying as $\lambda$ approaches 1.0 and narrower distributions of $\lambda$ values, although the number of identified events is small. TALL, OSBS and ORNL all have a large number of events during the modelling period, with a wider range of decay rates. ONAQ, CPER and GUAN have fewer events and longer stretches of the drying period, with relatively slower decay. The histograms of UNDE and SCBI appear to be flatter, possibly due to the variations in their decay patterns throughout the year.

The result also shows that there is a moderate negative correlation between the quantiles of $\lambda$ and MAP, indicating that wetter sites, e.g., OSBS (MAP = 1302 mm), SCBI (MAP = 1126 mm) and ORNL (MAP = 1340 mm), tend to have faster rates of soil moisture decay, and vice versa. This may be interpreted as drier sites having consistently slower drying driven by predominantly evapotranspiration and water draining through small pores, and wetter sites having more rainfall events with sufficient intensity, and wet antecedent conditions to initiate preferential vertical and lateral flows^38–40^. There is a weak negative correlation between the sand content and $\lambda$, and a weak positive correlation between both silt and clay and $\lambda$, aligning with the assertion that drainage is more rapid in coarser textured soils, such as in OSBS.

### Multi-depth analysis of travel times in the soil profile

**Fig. 6:**
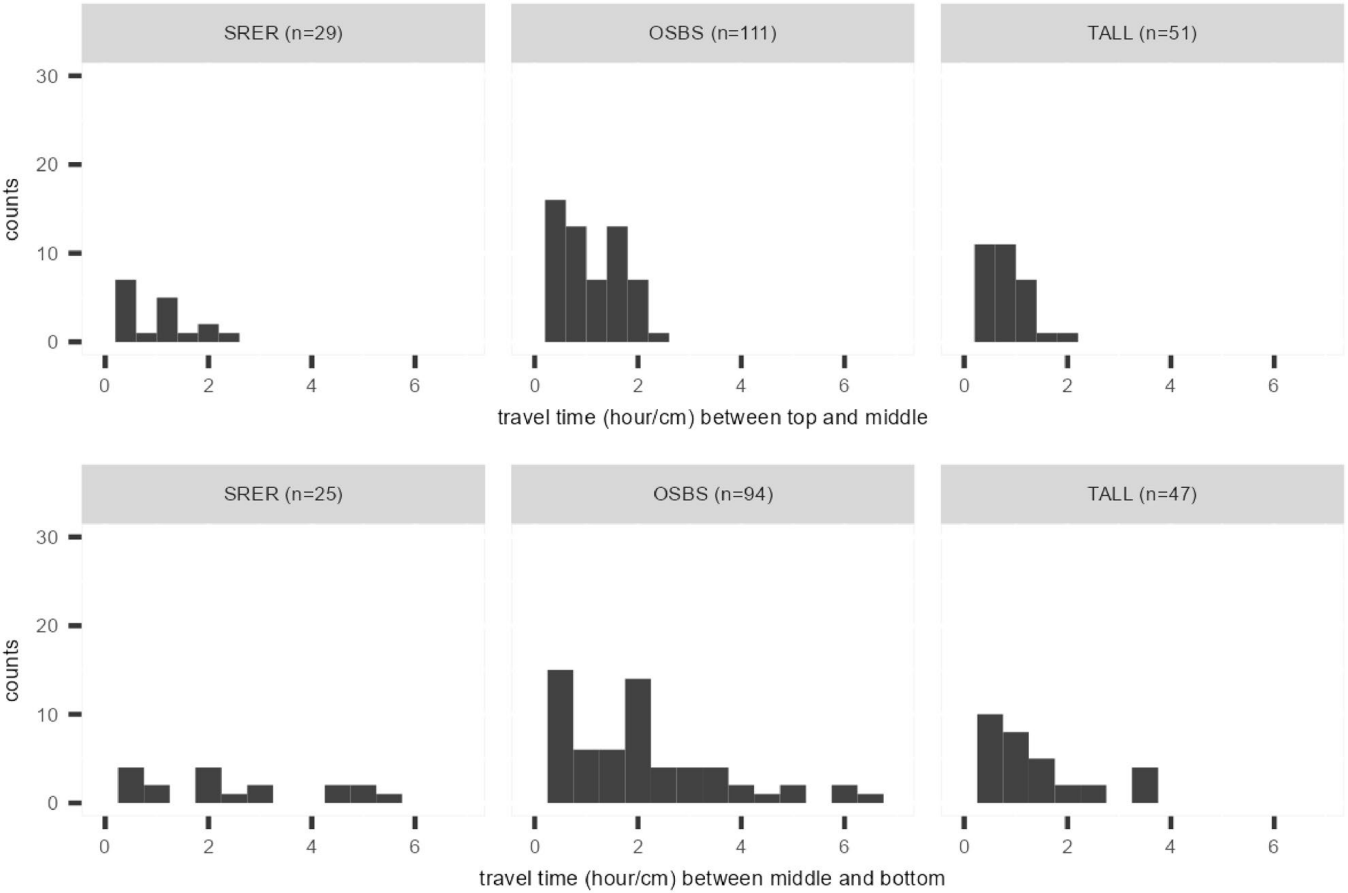
Histograms of travel time between the peaks detected in the soil moisture time series from the upper and mid levels (top), and mid and lower levels (bottom). The letter *n* stands for the number of travel times consisting of the histogram.

Figure 6 presents the histograms of the travel times between the upper- and mid-layer and the mid- and lower-layer from SRER, OSBS and TALL. The majority of the histograms display a pattern that indicates the dominance of shorter travel times (i.e. more rapid flow of water between the depths). The histograms demonstrate that travel times are longer in the lower layers. Within a field site, this may be a result of a decrease of hydraulic conductivity with depth^34,41^, or it may be related to more complex features, e.g., effects of horizonation^41,42^. There are more variations between sites which can potentially affect travel times, such as different climate conditions, topographic features, and soil and water chemical compositions^42^. In this case, TALL has the shortest travel times in both the upper and lower horizon, while the travel times of SRER and OSBS cover a wider range of values. Although the travel times of OSBS tend to concentrate between 0 to 2 hours/cm, those of SRER tend to be more uniform. These differences may be related to the climate and soil composition of the field sites. The combination of sandy soils at TALL and OSBS (90.7% and 97% respectively), and high rainfall (MAPs are 1328 mm and 1302 mm respectively), lead to shorter travel times than at SRER (78.8% sand and a MAP of 346 mm).

The results above demonstrate that the histograms of the estimated parameters and the travel times have the potential to distinguish different features across the field sites. These features can be explained by the soil contents, climates and vegetation of the field sites. In other words, the histograms (or other summary statistics of the distribution) can reflect some interesting properties of the soil in the field sites, and they may be considered jointly with the distributions of the drydown parameters as a multidimensional metric unique to the soil in a particular field site. Comparing these metrics provides a way of understanding the soil across sites and potentially a way of investigating the changes in the soil when future data become available.

## Discussion and conclusions

The analysis presented here provides a proof-of-concept for autonomously extracting physically interpretable information from time series data regarding soil properties and climatic regime. The development of automated approaches is necessary for dealing with the growing volumes of data being collected and in delivering more ubiquitous cost-effective soil monitoring. This will become increasingly important as soil monitoring stations are established, such as the NEON sites used as illustrations in this study and the soil lighthouses being developed within the European Union^43^. Autonomous data processing has the potential to add significant value to data sets, allowing data to be examined in almost real-time, anomalies identified and early detection of change facilitated. This helps mitigate the risk that large amounts of soil data will be collected but not utilised to their full potential.

In this study, we presented a changepoint-based method for dynamically and autonomously extracting information from individual soil moisture sensor time series, and from sensors at multiple depths. Our approach to extracting information from the drydown curves allows the investigation of temporal variations of the exponential decay model parameters in fine time resolutions, via visualising the model output as time series and summarising the variations of the estimated parameters for a given time period as distributions. The result from analysing the data from nine NEON field sites with contrasting soils and climates demonstrated, as anticipated, that the distributions of model parameters vary based on these factors. Across the sites, significant relationships were found between the asymptotic parameter $\alpha _0$ and MAT, and the decay rate $\lambda$ and the MAP. Whereas only moderate relationships were observed between the extracted parameters and soil composition. Climatic seasonality can also be observed in the time series plots and the distributions of the parameters extracted.

### Potential of the method to separate the two phases of drydown

As discussed in Section 2.2, with the availability of high frequency observations, different stages of the drydown process can potentially be observed, and hence the potential is there to estimate the decay rate during the drainage-dominated phase and the evapotranspiration-dominated phase separately. Although our changepoint-based method was developed for identifying the peaks in soil moisture time series, it can potentially be extended to detecting the changes in the drydown rates. Since we allow all parameters in the exponential decay model to vary from segment to segment, a change in the decay rate can be detected if it makes a significant difference to the likelihood.

**Fig. 7:**
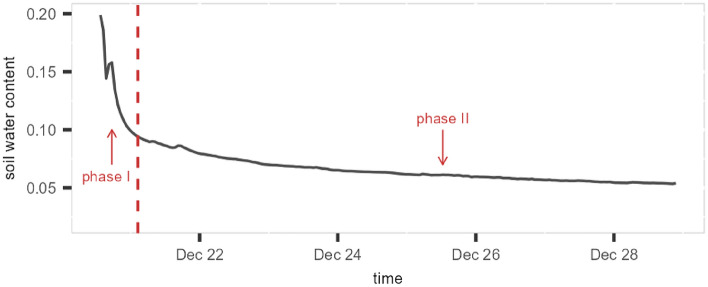
An example of using changepoint detection to identify the two phases of soil moisture decay, with phase I showing a faster decay rate ($\lambda _{1} = 0.86$) than phase II ($\lambda _{1} = 0.98$).

Here, we provide a toy example of detecting a change in the decay rate. We applied the same method to a decreasing segment from the soil moisture time series of site OSBS. The only change made here was lowering the threshold of the jump size to 0, so that the time point with a parameter change but no obvious jump can also be detected. In this case, using a relatively large penalty, a changepoint was detected at 14 hours after the beginning of the drydown (see Fig. 7). It divided the segment into two parts, with the first part showing a faster decay rate ($\lambda _{1} = 0.86$) than the second part ($\lambda _{1} = 0.98$). This changepoint could potentially be the transition point from the drainage-dominated phase to the ET-dominated phase, but it is difficult to verify. To validate such a method would require lab or field experiments, possibly also theoretical statistical work, to provide information regarding the location of the transition point. Nonetheless, this toy example demonstrates the potential of the changepoint-based method to distinguish the two phases of the drydown process.

### Potential of the method to investigate long-term changes

We hypothesise that, given enough data over time, it would be possible to form a characteristic or representative distribution of the model parameters for a period of time at a given location. Detecting temporal changes in these characteristic distributions would potentially indicate a change to the soil water regime, due to a longer-term change in climate, vegetation or soil properties. For example, soil degradation events, such as compaction, might be detectable as an increase in expected travel times between different soil depths^44^. Improvements to soil health may also be detectable. For example, increases in soil organic matter content may alter distributions in $\lambda$ as the capacity of soils to hold water increases; improvements to soil structure over time with changing crop practices could result in decreased travel times indicating improved infiltration^45^. The ability to automatically detect changes in soil drydown characteristics over time would open up the potential for real-time monitoring and automated soil diagnostics.

**Fig. 8:**
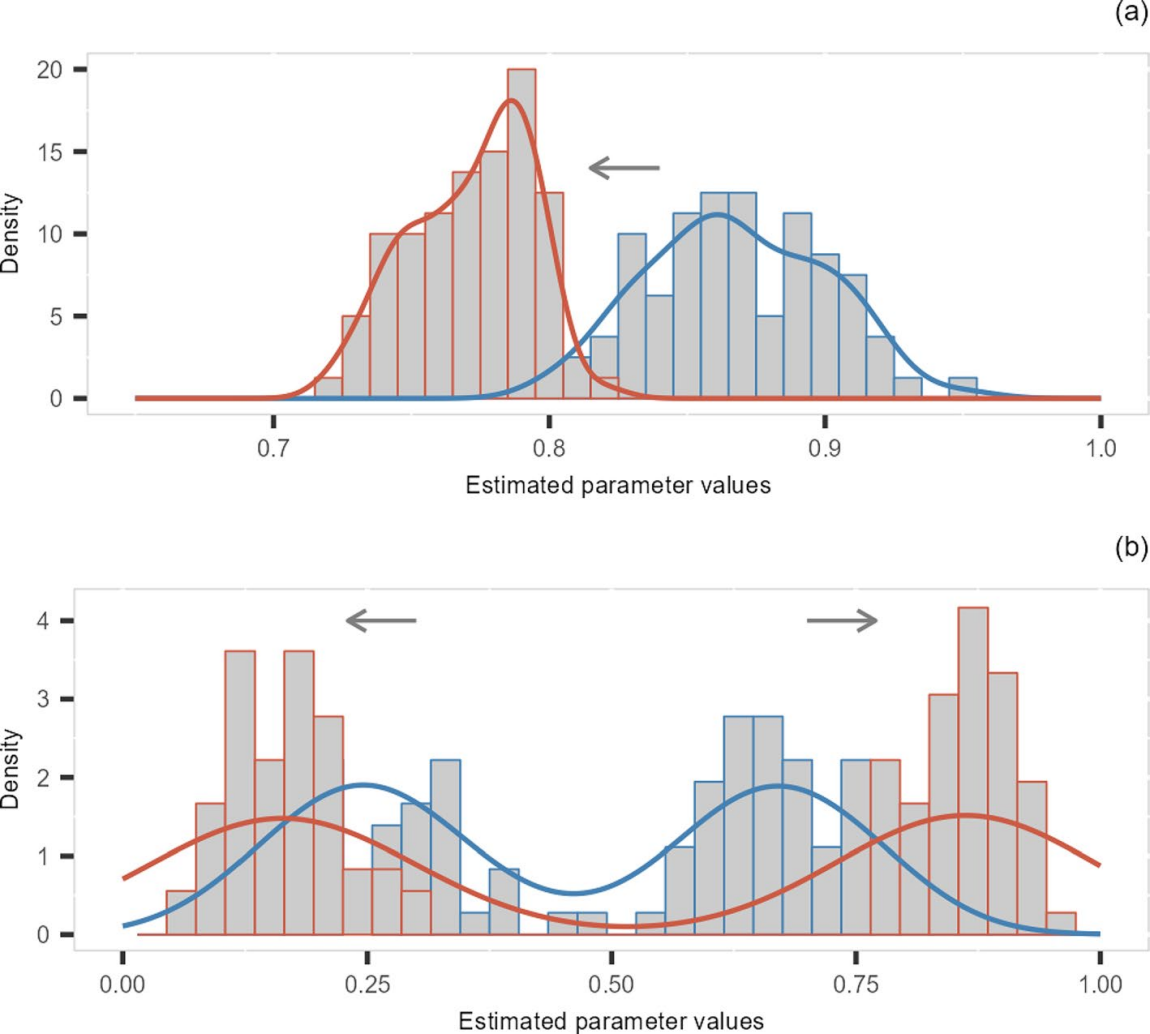
Examples of the changes of the characteristic distributions. (**a**) Histograms and density curves representing a mean shift from the red to blue. (**b**) Histograms and density curves representing a transition to a more extreme bimodal distribution.

To further illustrate the potential of the method to investigate long-term changes, two examples are given in Fig. 8. Assume that the characteristic distribution of the estimated parameter can be represented by a histogram or a density curve. Potential changes in soil drydown characteristics would then correspond to the changes in the range or shape of the distributions. For example, the change may be a mean shift from the blue histogram to the red histogram as illustrated in panel (a), or it may be a transition into a more extreme bimodal distribution, as illustrated by the shift from the blue histogram to the red histogram in panel (b). It is possible to carry out statistical tests (e.g., Kolmogorov–Smirnov test) to determine whether the distributions before and after are significantly different, in order to conclude whether there is a change or not.

We speculate that it may also be informative to combine multiple temporal measures to form a joint metric, such that it encompasses multiple dimensions of soil health^2^. For example, combining soil moisture time series data with $\textrm{CO}_{2}$ and temperature time series data to derive a joint characteristic distribution of some key parameters. This might help in diagnosing the nature of change, for example, helping discern whether a change is attributable to the soil structure or climate. In addition to the investigation of temporal variation and distributional change of drydown parameters, the proposed method may also benefit the analyses which require the information on the timing and frequency of the soil moisture drydown. The method may be further extended to include the changes within a drying segment, i.e., the identification of different drydown stages. This will provide important information to analyses such as the “bottom up” method of estimating precipitation from soil moisture time series^46,47^.

Despite being automatic and easy to implement, the changepoint-based approach has some limitations. The method requires as input a pre-determined type of model to fit the drydown curve. This lack of flexibility means that the model may not be able to describe the patterns in the time series during the saturated period appropriately, as the time series during the saturated period do not usually display an exponential decay pattern. It can also encounter problems when there exist slow wetting periods that last for more than just a few hours. This problem can be more severe in some sites than others, depending on the rainfall pattern, soil texture and other environmental factors of the sites. As an exponential decay model is incorrect for these periods, the estimated model parameters often come with high uncertainty, or a lack of convergence. These estimations need to be excluded from the post analysis, resulting in a smaller sample size in the post changepoint analysis. To address this problem, one future extension would be to allow different types of changepoints in the model. For example, one can define the changepoint to be either the time when the dyring starts or the time when the wetting starts, and specify two candidate models, a drydown model and a wetting model, to model both processes. We then let the changepoint detection algorithm select the more suitable model for the segment. Bayesian online changepoint detection approach^48^ provides a potential framework. However, the extension can be challenging due to the complexity in the soil moisture dynamics.

The changepoint-based method can be extended to other soil time series that respond to discrete events (e.g., rainfall), as long as the response can be described using the same type of model. However, it does not apply to other soil processes that are subject to more continuous or complex perturbations straightforwardly. Therefore, further development of the approach or alternative methods may be required to analyse time series such as soil $\textrm{CO}_{2}$ and soil microbial activity.

## Supplementary Information

Supplementary Information.

## Data Availability

The changepoint detection algorithm and the analysis of the soil moisture time series are implemented in R (version 4.3.1). The code were developed by the authors and can be accessed from the GitHub repository https://github.com/GMY2018/Changepoint4soil. The soil moisture data used in the analysis are publicly available from the United States National Ecological Observation Network (NEON, https://data.neonscience.org/). In particular, the time series data can be downloaded from https://data.neonscience.org/data-products/ DP1.00094.001.
