# Supplementary material for: A changepoint approach to automated estimation of soil moisture drydown parameters from time series data
Source: Sci Rep. 2025 Dec 2;15:42970. doi: 10.1038/s41598-025-27067-w (PMC12673061; doi:10.1038/s41598-025-27067-w)
Supplement: Supplementary file 1 — Supplementary Information. [file 41598_2025_27067_MOESM1_ESM.pdf]

Supplemental file to the manuscript "A changepoint approach to automated estimation of soil moisture drydown parameters from time series data"

1 Introduction of the changepoint detection method

Here we provide a brief introduction to the changepoint analysis method for soil moisture time series[1]. Consider the sudden increase of soil water content as a "change" to the exponential decay process that would have continued without disturbance. If we define the time point right before the i-th peak as a "changepoint", then the segment between the i-th peak and the $(i+1)$-th peak, i.e., the segment between two adjacent changepoints, τ_i and τ_{i+1}, represents approximately the drydown process and can be modelled using an exponential decay model, as

$$\begin{aligned} \theta_t &= \alpha_{0i} + \alpha_{1i}\,\lambda_i^{(t-\tau_i)} + \epsilon_t \\ &= \alpha_{0i} + \alpha_{1i}\,\exp(-\exp(\gamma_i))^{(t-\tau_i)} + \epsilon_t \\ \epsilon_t &\sim \mathcal{N}(0,\sigma^2)\ , \end{aligned} \tag{1}$$

where the re-parameterisation $\lambda_i = \exp(-\exp(\gamma_i))$ is to remove the constraint on parameter values. Following this idea, the problem of identifying the drydown curves and fitting the drydown models can be reframed as a changepoint detection problem with simultaneous parameter estimation. Asssume there are k changepoints between time 0 and time n, i.e., $0 = \tau_0 < \tau_1 < \cdots < \tau_k < \tau_{k+1} = n$. Detecting the changepoints translates to minimising the overall penalised cost function over $\tau_1, \cdots, \tau_k$, i.e., to find

$$F(n) = \min_{0=\tau_0<\cdots<\tau_{k+1}=n} \sum_{i=0}^{k} \mathcal{C}(\theta_{(\tau_i+1):\tau_{i+1}}) + \nu k\ , \tag{2}$$

where $\mathcal{C}(\theta_{(\tau_i+1):\tau_{i+1}})$ is a cost function of the segment $\theta_{(\tau_i+1)}, \cdots, \theta_{\tau_{i+1}}$. A common choice of the cost function is two times the negative log-likelihood of the exponential decay model (1) fitted to the segment. This can be calculated using nonlinear least squares estimation of the model (1). The number of changepoints k is used to penalise the model complexity and a penalty parameter ν is used to adjust the strength of the penalty. This is the only parameter that requires manual selection. The selection of penalty parameter in penalised optimisation problems typically involves a grid search over a series of ν values to find the one that produces minimises or maximises certain selection criterion, e.g., squared prediction errors and cross-validation scores[2]. Specific selection methods devoted to changepoint problems were introduced in literature[3,4,5]. Here, we adopt the grid search method[6] to determine the ν value.

The penalised exact linear time (PELT) method[5] is used to solve the optimisation problem (2). The algorithm starts with the recursive computation of the overall cost function of data up to

time point s

$$F(s) = \min_{\boldsymbol{\tau} \in \mathcal{T}_s} \left\{ \sum_{i=0}^{m} \mathcal{C}(\theta_{(\tau_i+1):\tau_{i+1}}) + \nu\, m \right\} = \min_{0 \le \tau < s} \left\{ F(\tau) + \mathcal{C}(\theta_{(\tau+1):s}) + \nu \right\} ,$$

for $s = 1, \cdots, n$. Instead of searching through all possible time points $0 \le \tau < s$ for the optimal solution to τ, the algorithm prunes the time points that can never be the last optimal changepoint prior to time s based on an inequality of the overall cost and the cost of the last segment, and only searches within a subset of all possible time points to reduce the computational cost to linear in the length of the time series. Thus it reduces the computation time.

The algorithm returns the estimated changepoints, $0 < \hat{\tau}_1 < \cdots < \hat{\tau}_k < n$, and the estimated parameters for each exponential decay segment, $\hat{\alpha}_{0i}$, $\hat{\alpha}_{1i}$ and $\hat{\lambda}_i$ (from $\hat{\lambda}_i = \exp(-\exp(\hat{\gamma}_i))$), $i = 1, \cdots, k$. Computing the distributions and summary statistics of the estimated parameters from different soil moisture time series enables the extraction of metrics that characterise the hydraulic properties of soils in different field sites. Visualising the model parameters as a time series helps to reveal the temporal patterns of the drying process.

The proposed method can be implemented in R [7] using code developed for this particular problem [1]. In particular, the exponential decay model parameters are estimated using the nonlinear least square optimisation algorithm in package `nlmrt` [8].

The changepoint-based method offers an automatic way to model a large number of soil moisture time series data. It requires little data pre-processing and can be applied to the time series data directly given the tuning parameter. However, the method requires a complete time series without missing observations. Therefore, it cannot be applied to time series with large missing gaps which are impossible to interpolate without relevant information. It may encounter problems when it is applied to a time series consisting primarily of saturated or frozen periods, as soil moisture behaves differently during these periods.

2 Influence of the rewetting on drydown estimation

In the main manuscript, we described that the segment between two adjacent changepoints (i.e., soil moisture peaks) are *approximately* a drydown segment. The reason that it may not be an exact drydown curve is because the curve may contain part of the rewetting segment, if the rewetting process happened over a longer period than just one or two hours. This can happen in some field sites during certain time of the year where the rainfall patterns and the soil structures are more prone to a slower rewetting. This could have some influence on identifying the drydown curves and estimating the drydown parameters.

The biggest impact seems to be the higher uncertainty in parameter estimation. Sometimes the estimation from the nonlinear least square (NLS) optimisation may not converge, or it may hit the boundaries set for the parameters. Both result in very high standard errors in the estimation. Segments with very high uncertainty need to be excluded from the analysis, such as the histograms and the correlation table, as mentioned in section 2.3 of the main manuscript. From what we observed in the analysis of the nine NEON sites, time series from sites UNDE, CPER and ONAQ, which are located in the colder areas of the U.S., are more prone to this problem; whereas those from the rest of the six sites in warmer areas are less problematic. Another

impact is the mis-estimation of the decay parameter (more likely to be an under-estimation), due to the involvement of the observations from the slow rewetting process. Both the scale of the increase and the proportion of observations belong to the rewetting period appear to have an impact on the NLS estimation. For most of the segments where majority of the observations are from the drying period, the mis-estimation will not be severe.

The left panel of Figure S1 shows an example from site ONAQ, where the slower rewetting period and following drying period were separated by the changepoint detection algorithm and were estimated separately (see the red curves and the red vertical line). This resulted in the first segment being discarded due to boundary solutions, and an slight under-estimation of the decay parameter, as compared to the estimation based on the drying part of the curve only (see the blue curve). In particular, the estimation based on the segmentation is 0.9925, which corresponds to an e-folding decay time scale of 5.50 days (132 hours); the estimation based on the decreasing only curve is 0.9918, or 5.04 days (121 hours) for the e-folding decay time scale. The right panel shows an example from site TALL, where the rewetting process was included in the drying process. However, as there are only four observations from the rewetting process, their impact on the estimation was small. In this case, the red curve (fitted using the segmentation from the changepoint algorithm) and the blue curve (fitted using the only the drying part of the time series) are very close. The estimated decay rates are 0.9933 and 0.9937 respectively, and the e-folding decay time scales are 1.50 and 1.54 days respectively.

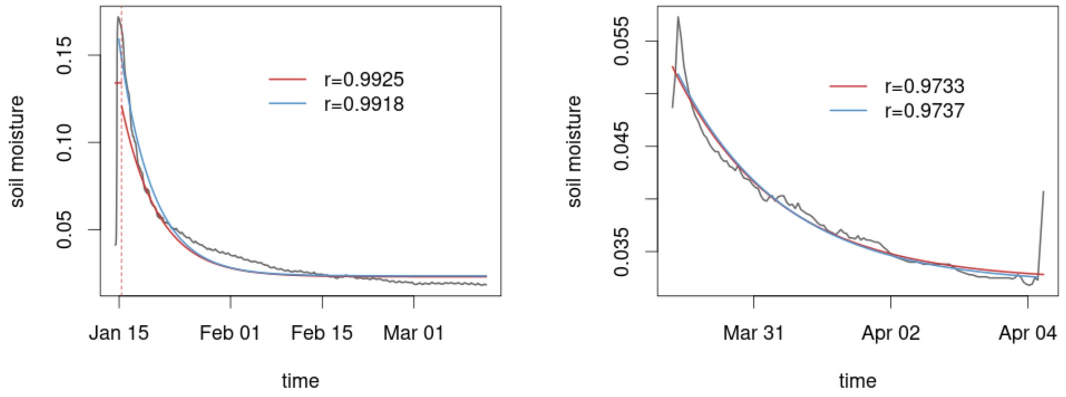

Figure S1: An example of the rewetting and drying process being segmented and resulting in the first segment being discarded (left), and an example of the rewetting process being included in the drying process and resulting in an uncer-estimation of the decay rate.

3 Additional information and figures of modelling result

Here we present maps of annual mean temperature and soil texture with marked locations of the nine NEON field sites in Figure S2, and Table S1 which summarises the estimated asymptotic parameter α_0 and the estimated decay parameter γ. The maps in Figure S2 were generated in R[7] (version 4.4.1, https://cran.r-project.org/bin/windows/base/old/4.4.1/) using SoilGrids250m data[9]. Figures S3 to S11 show the result from the changepoint-based analysis for the nine NEON field sites as time series plot and piecewise constant time series plot.

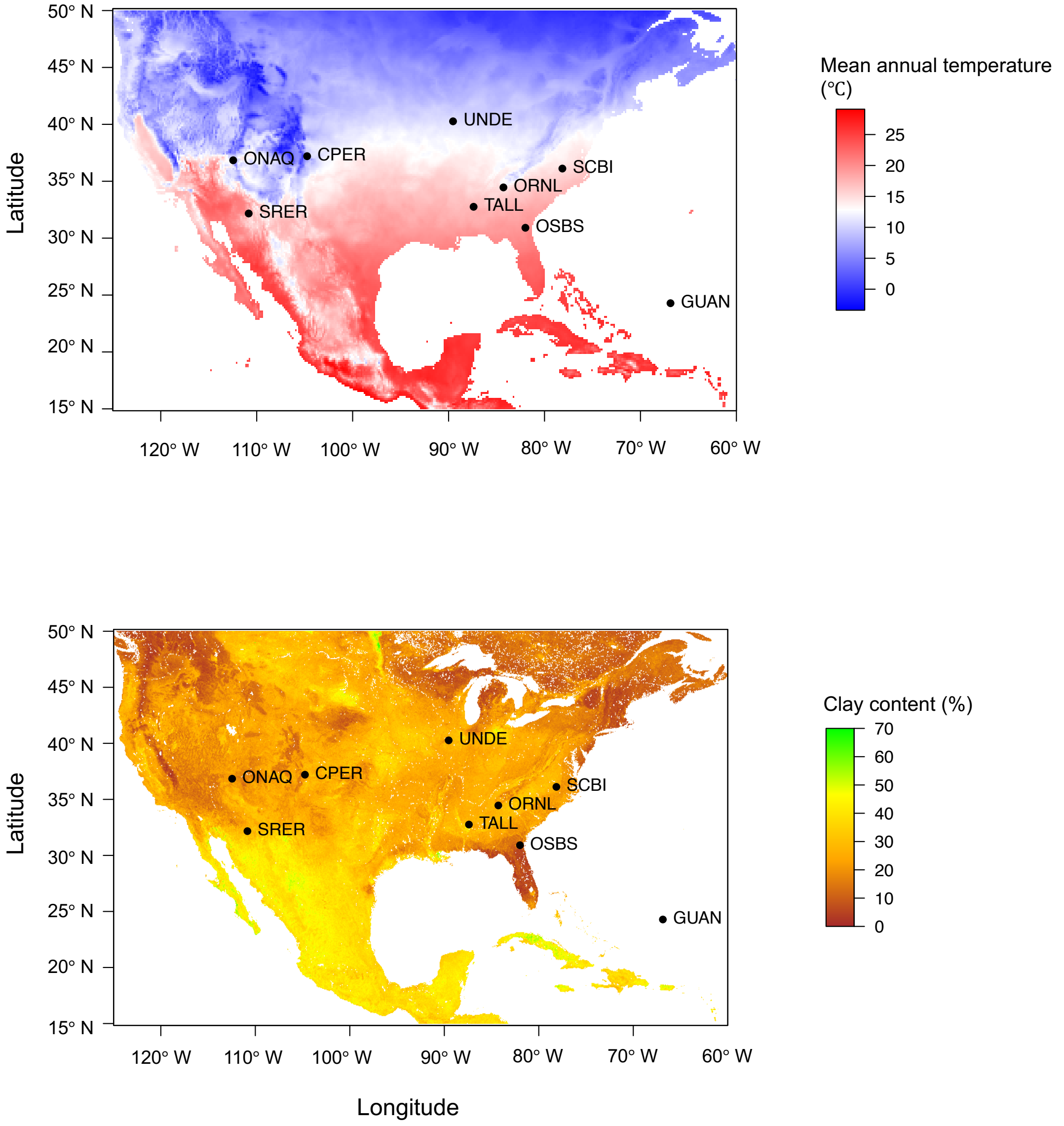

Figure S2: Maps showing the distribution of the study sites overlaid on mean annual temperature (top) and clay content (bottom). Software: R[7] version 4.4.1 (https://cran.r-project.org/bin/windows/base/old/4.4.1/). Data source: SoilGrids250m[9].

Table S1: Summary of the estimated asymptotic soil moisture α_0 and decay rate λ after removing boundary solutions and estimations with large standard errors.

	$\hat{\alpha}_0$					$\hat{\lambda}$				
	Min.	1st Qu.	Median	3rd Qu.	Max.	Min.	1st Qu.	Median	3rd Qu.	Max.
SRER	0.0206	0.0233	0.0304	0.0470	0.0688	0.8835	0.9771	0.9901	0.9947	0.9984
TALL	0.0005	0.0165	0.0327	0.0413	0.0552	0.8933	0.9670	0.9781	0.9899	0.9985
OSBS	0.0155	0.0424	0.0533	0.0666	0.1127	0.6972	0.9255	0.9611	0.9801	0.9942
UNDE	0.0811	0.1313	0.1431	0.1642	0.1888	0.8561	0.9359	0.9640	0.9810	0.9976
CPER	0.0001	0.0088	0.0102	0.0467	0.1747	0.9684	0.9784	0.9848	0.9926	0.9970
SCBI	0.0040	0.0290	0.0668	0.0773	0.0997	0.8305	0.9487	0.9799	0.9918	0.9988
ONAQ	0.0116	0.0323	0.0590	0.0827	0.0995	0.9783	0.9895	0.9952	0.9965	0.9985
GUAN	0.0041	0.0101	0.0176	0.0217	0.0669	0.9144	0.9885	0.9913	0.9931	0.9983
ORNL	0.0652	0.0994	0.1249	0.1370	0.1502	0.8369	0.9564	0.9756	0.9896	0.9989

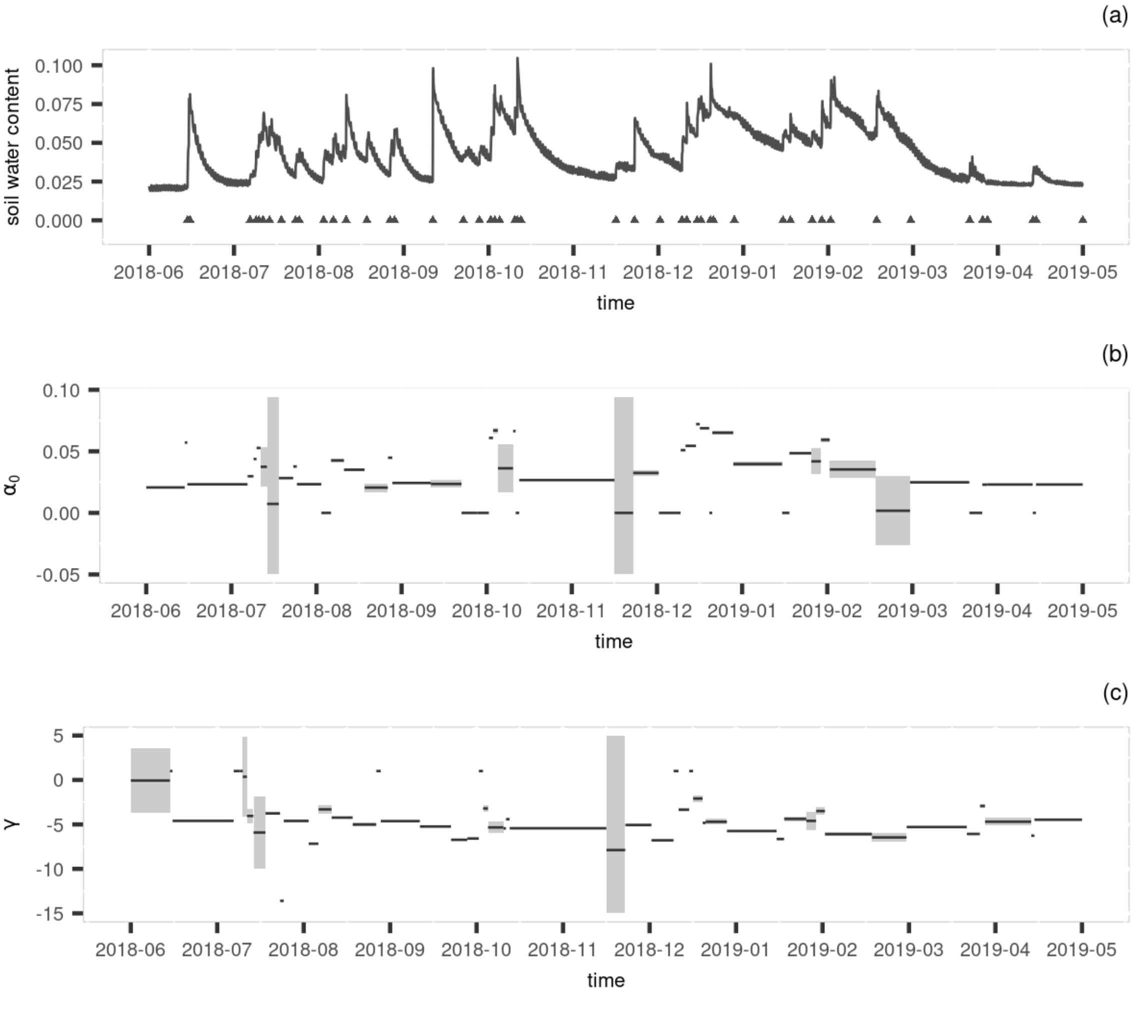

Figure S3: (a) The soil water content time series with the detected changepoints from field sites SRER. (b) The estimated asymptotic parameter α_0 plotted over time along with the confidence intervals (grey rectangles) (c) The estimated transformed decay parameter γ plotted over time along with the confidence intervals (grey rectangles).

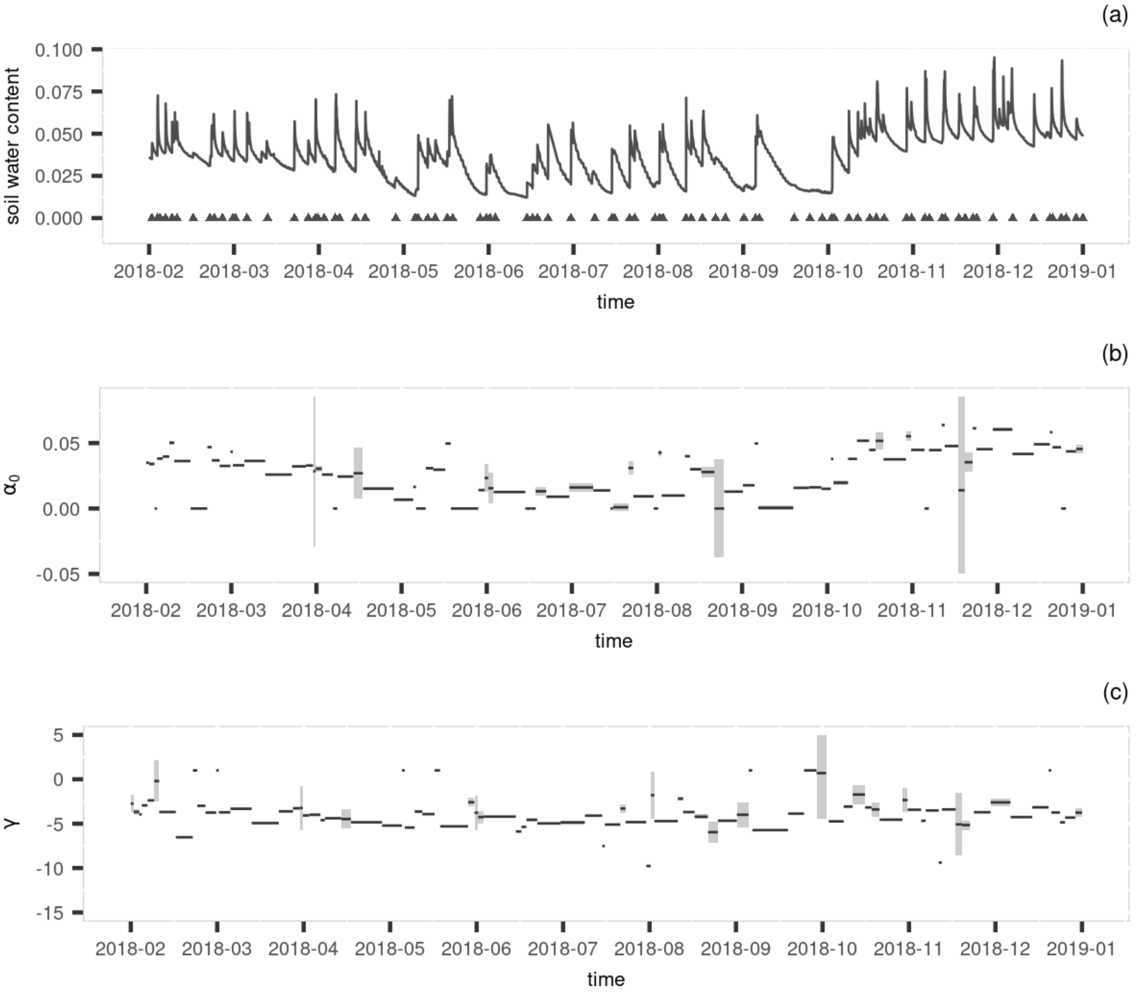

Figure S4: (a) The soil water content time series with the detected changepoints (black triangles) from site TALL. (b) The estimated asymptotic parameter α_0 plotted over time along with the confidence intervals (grey shaded rectangles) (c) The estimated transformed decay parameter γ plotted over time along with the confidence intervals (grey shaded rectangles).

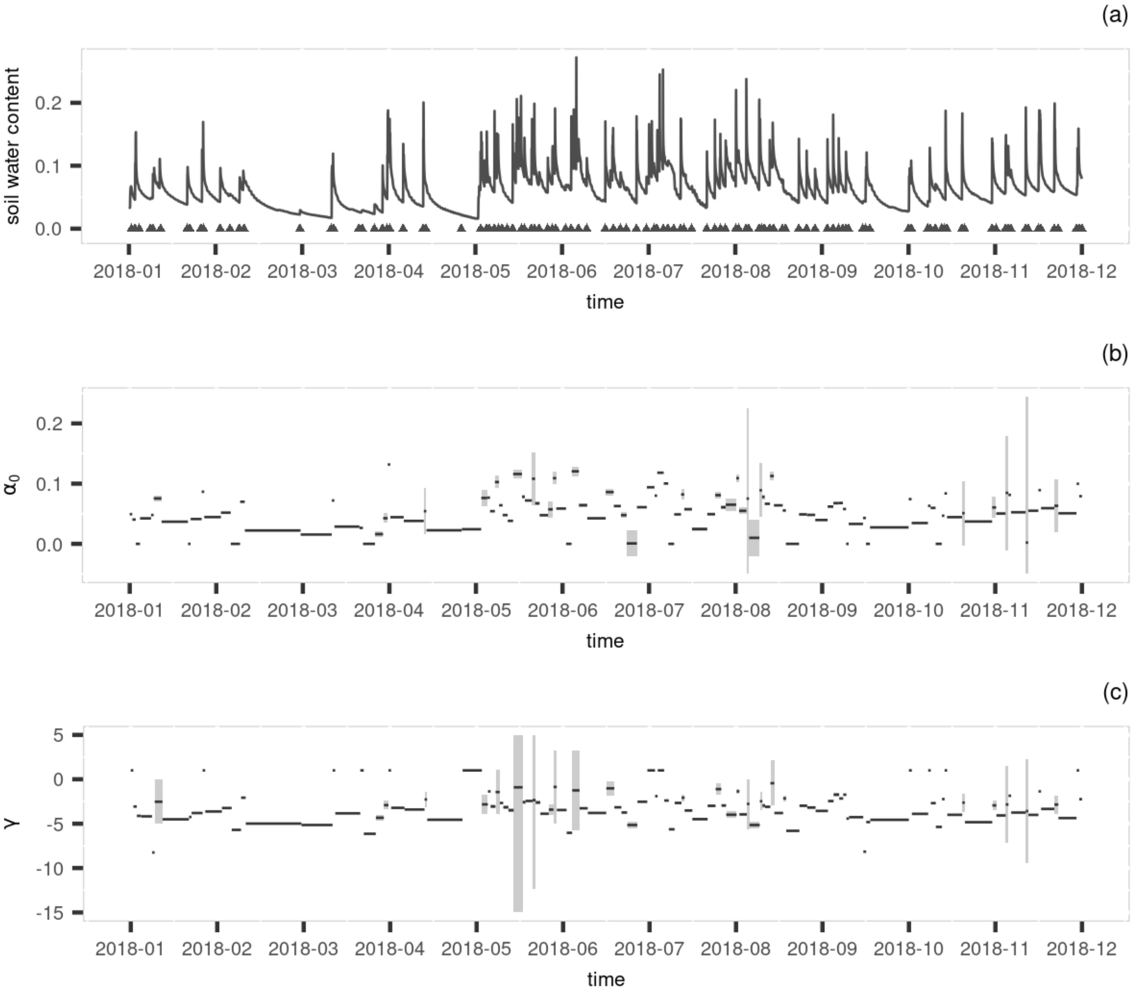

Figure S5: (a) The soil water content time series with the detected changepoints from field sites OSBS. (b) The estimated asymptotic parameter α_0 plotted over time along with the confidence intervals (grey rectangles) (c) The estimated transformed decay parameter γ plotted over time along with the confidence intervals (grey rectangles).

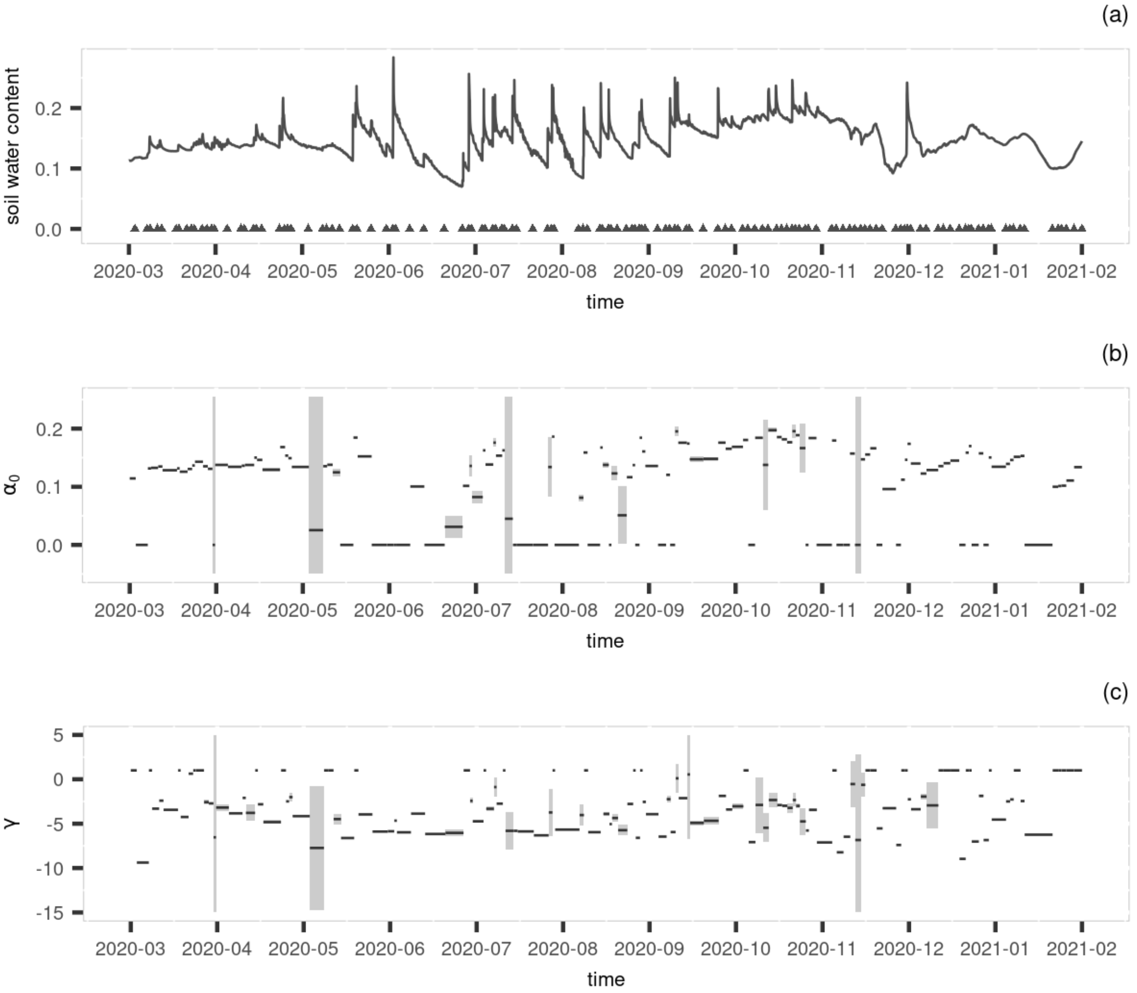

Figure S6: (a) The soil water content time series with the detected changepoints from field sites UNDE. (b) The estimated asymptotic parameter α_0 plotted over time along with the confidence intervals (grey rectangles) (c) The estimated transformed decay parameter γ plotted over time along with the confidence intervals (grey rectangles).

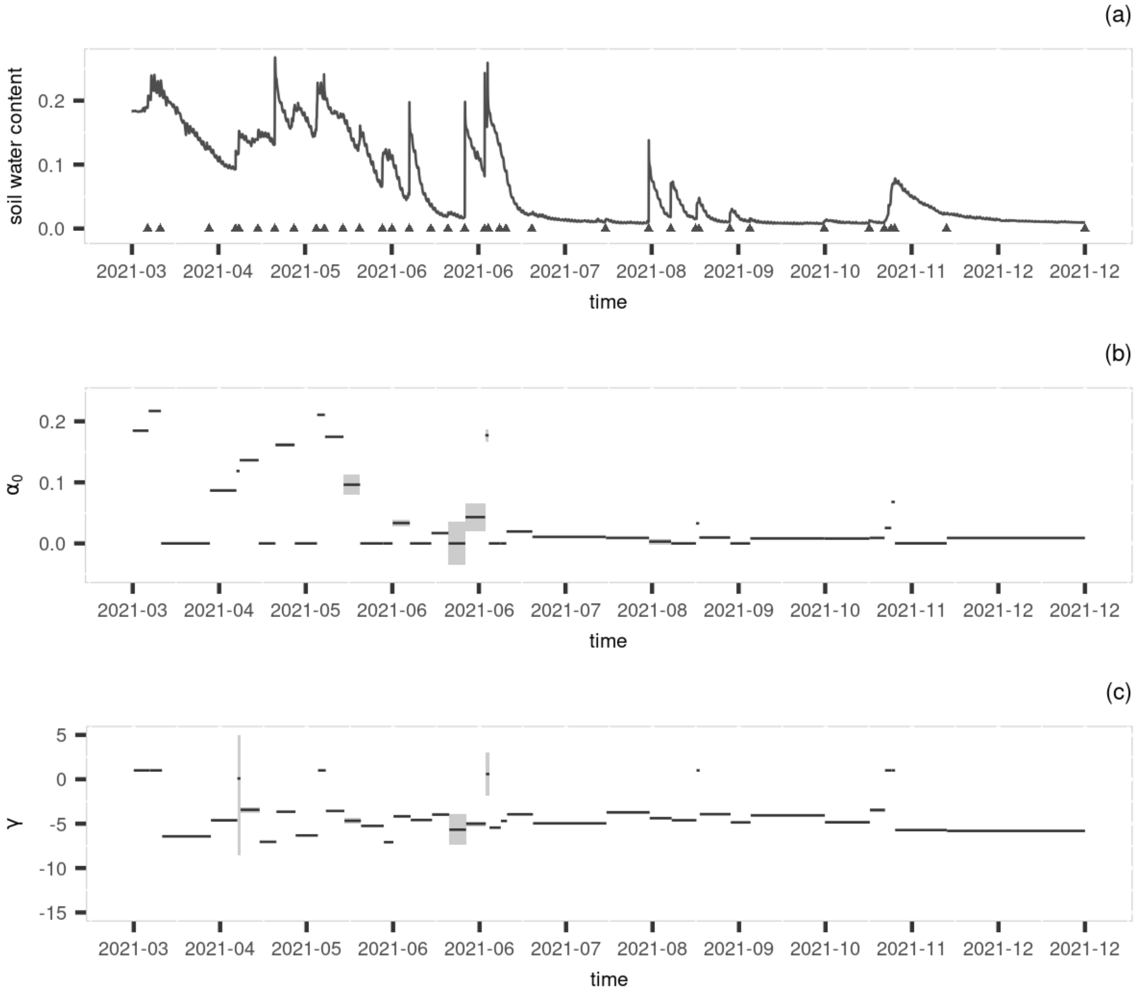

Figure S7: (a) The soil water content time series with the detected changepoints from field sites CPER. (b) The estimated asymptotic parameter α_0 plotted over time along with the confidence intervals (grey rectangles) (c) The estimated transformed decay parameter γ plotted over time along with the confidence intervals (grey rectangles).

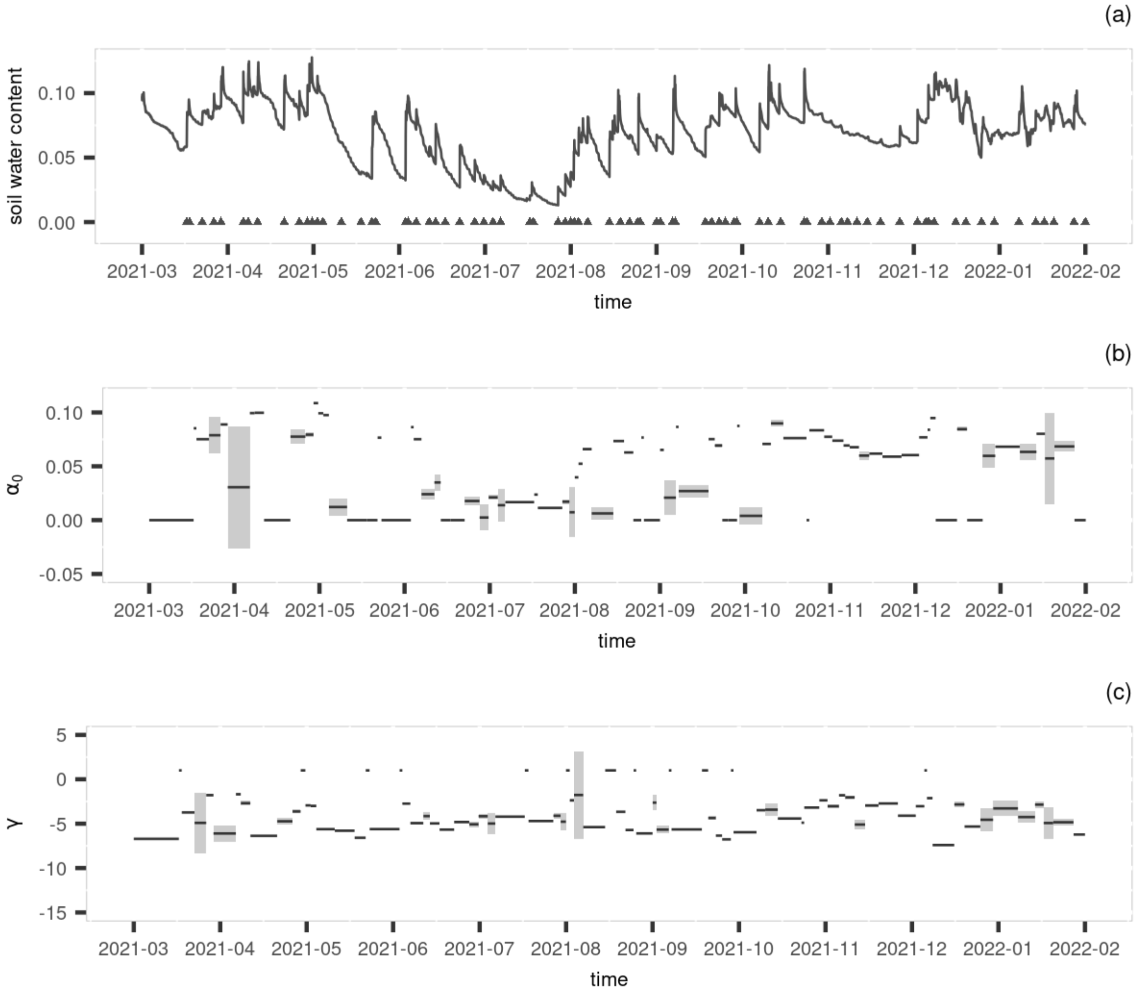

Figure S8: (a) The soil water content time series with the detected changepoints from field sites SCBI. (b) The estimated asymptotic parameter α_0 plotted over time along with the confidence intervals (grey rectangles) (c) The estimated transformed decay parameter γ plotted over time along with the confidence intervals (grey rectangles).

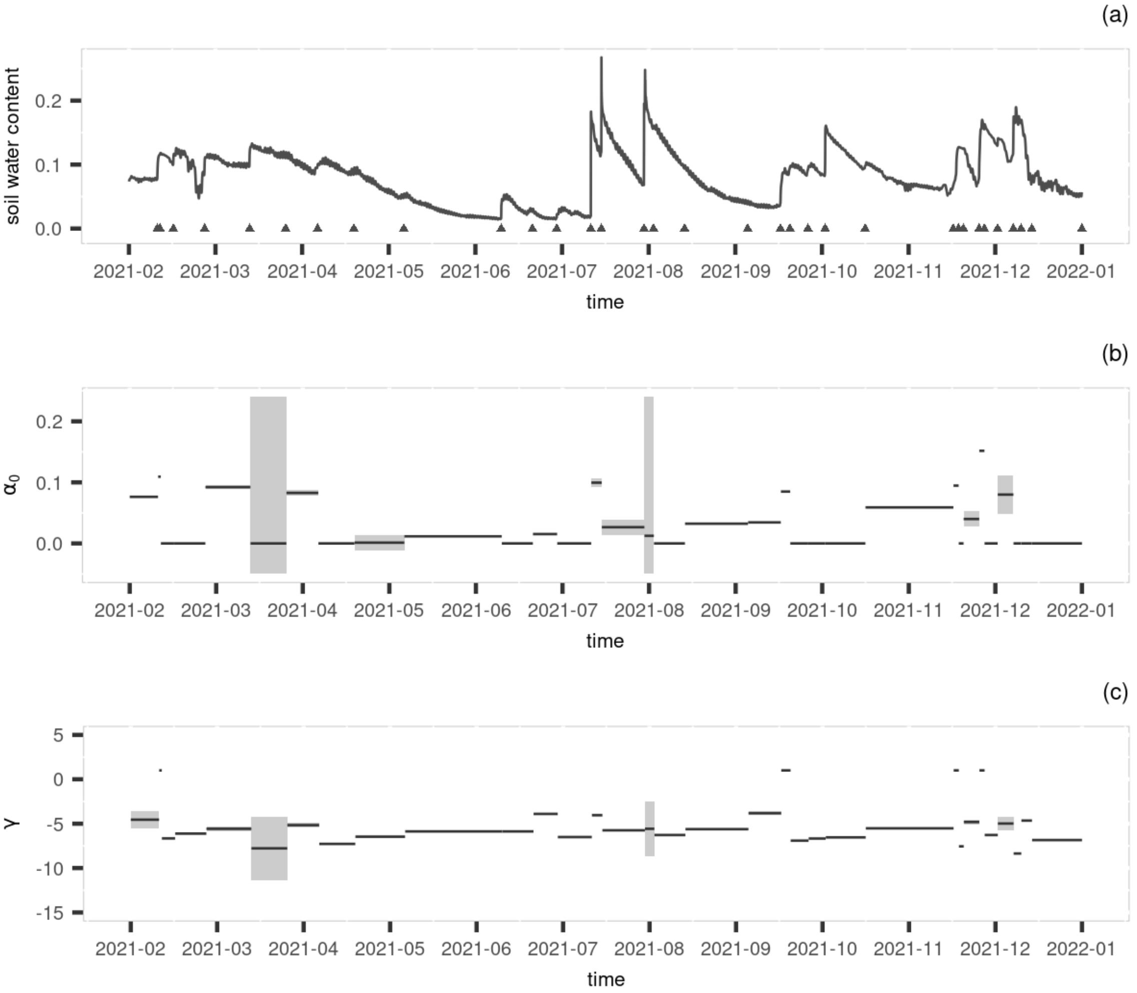

Figure S9: (a) The soil water content time series with the detected changepoints from field sites ONAQ. (b) The estimated asymptotic parameter α_0 plotted over time along with the confidence intervals (grey rectangles) (c) The estimated transformed decay parameter γ plotted over time along with the confidence intervals (grey rectangles).

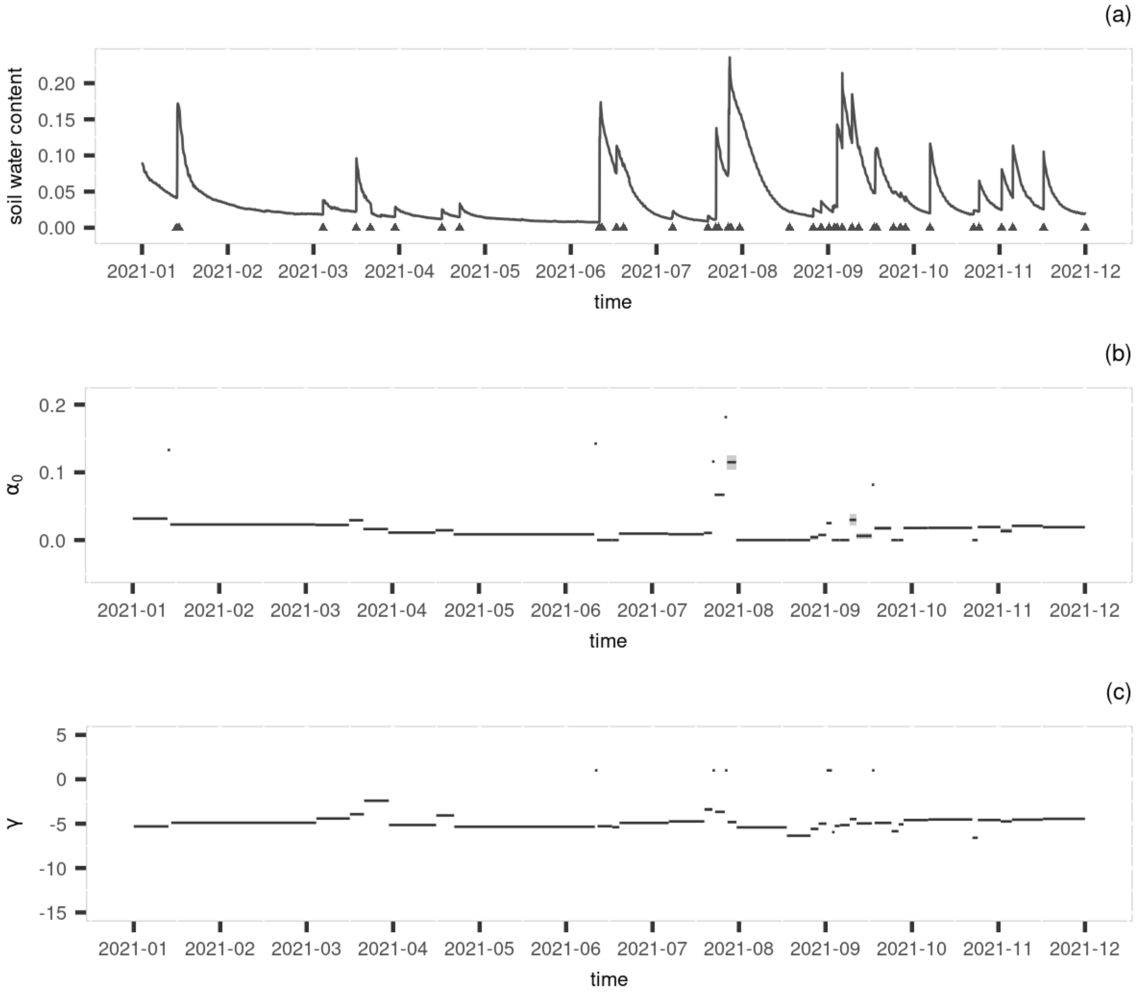

Figure S10: (a) The soil water content time series with the detected changepoints from field sites GUAN. (b) The estimated asymptotic parameter α_0 plotted over time along with the confidence intervals (grey rectangles) (c) The estimated transformed decay parameter γ plotted over time along with the confidence intervals (grey rectangles).

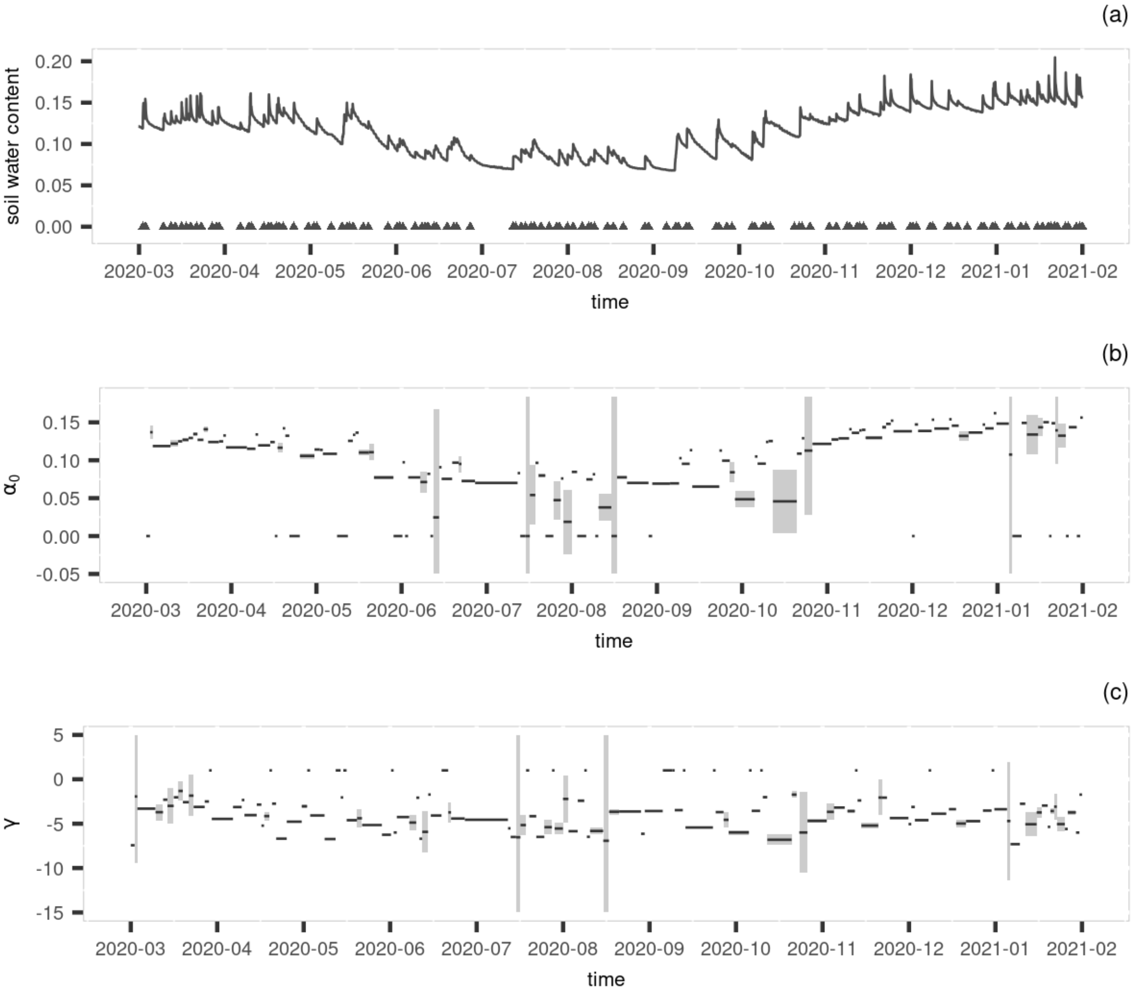

Figure S11: (a) The soil water content time series with the detected changepoints from field sites ORNL. (b) The estimated asymptotic parameter α_0 plotted over time along with the confidence intervals (grey rectangles) (c) The estimated transformed decay parameter γ plotted over time along with the confidence intervals (grey rectangles).

4 Additional information and figures of travel times

During the calculation of the travel times between peaks, a maximum delay of 24 hours is allowed between the top and middle levels and a maximum delay of 72 hours is allowed between the top and bottom levels in order to declare a match. The maximum delay is used to distinguish the peaks from different events. The threshold was determined based on a slow permeability of ~ 0.5 cm/hr, which is typical for sandy clay and silty clay soil[10,11,12]. This rate corresponds to a travel time of roughly 20 hours for every 10 cm. Hence we take 24 hours (i.e., a day) as the cutoff. We then set the maximum travel delay between the upper and lowest depth sensors as a multiple of the delay between upper and mid-depth, acknowledging that permeability generally decreases with depth over the profile[13]. Considering the soil composition of the three NEON sites (SRER, OSBS and TALL), these thresholds appear to be appropriate for all of them.

Below are figures showing the matching peaks from three layers from all the soil plots under study.

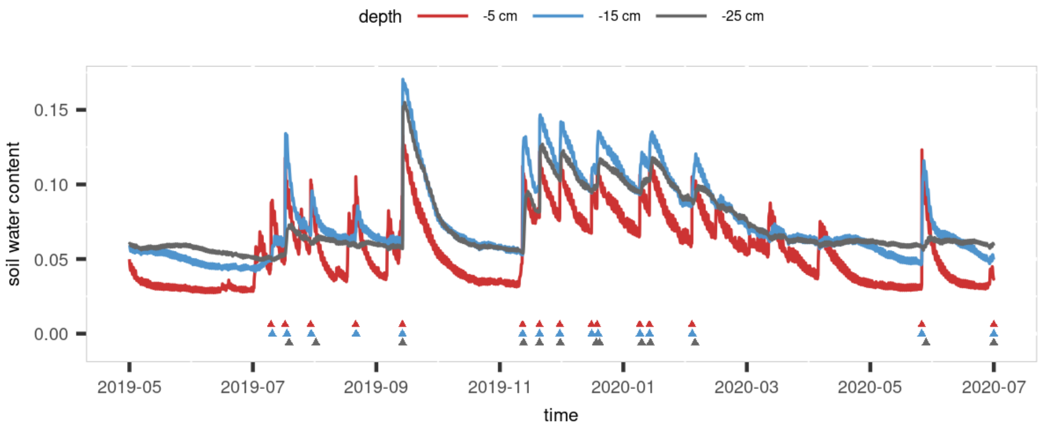

Figure S12: The soil water content time series recorded at -5 cm (red), -15 cm (blue) and -25 cm (grey) in location 2 of site SRER and the detected changepoints (coloured triangles) that has at least one match with a changepoint in the time series from another depth.

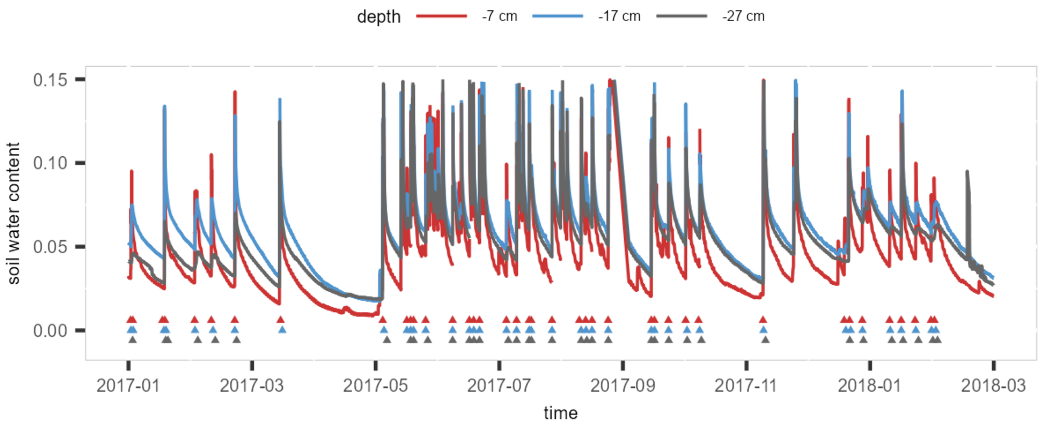

Figure S13: The soil water content time series recorded at -7 cm (red), -17 cm (blue) and -27 cm (grey) in location 1 of site OSBS and the detected changepoints (coloured triangles) that has at least one match with a changepoint in the time series from another depth.

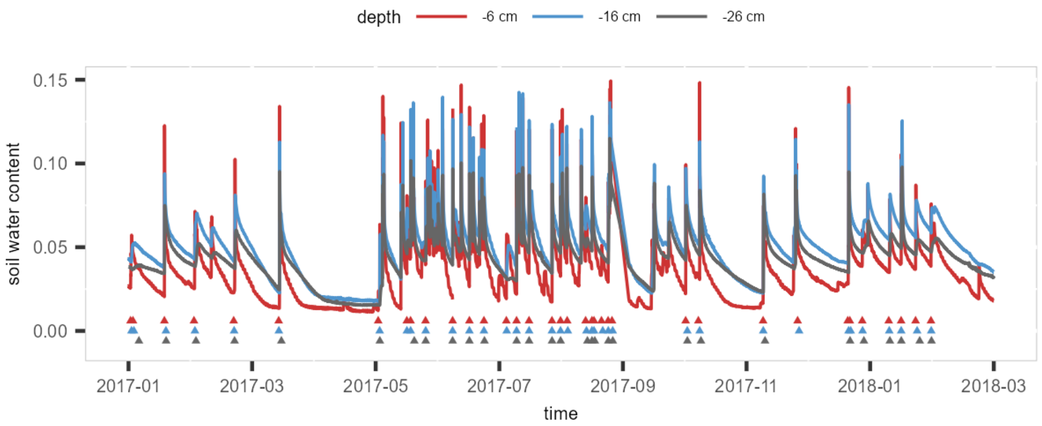

Figure S14: The soil water content time series recorded at -6 cm (red), -16 cm (blue) and -26 cm (grey) in location 2 of site OSBS and the detected changepoints (coloured triangles) that has at least one match with a changepoint in the time series from another depth.

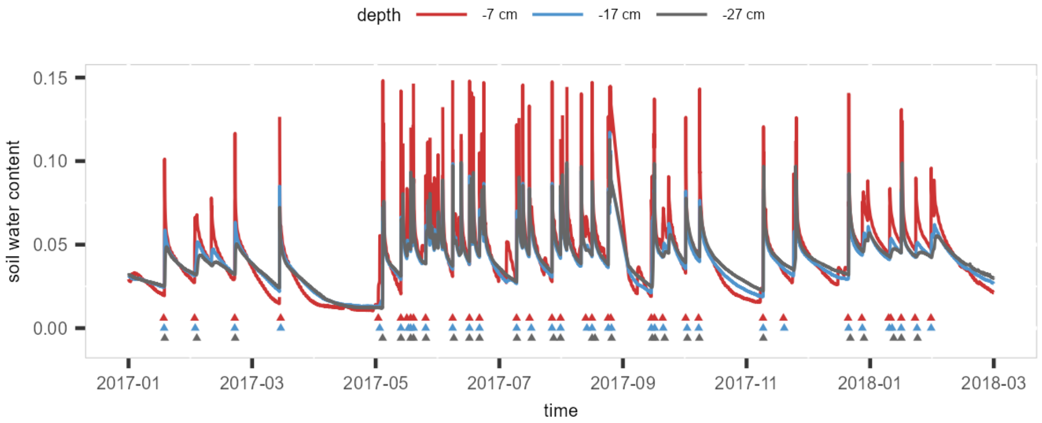

Figure S15: The soil water content time series recorded at -7 cm (red), -17 cm (blue) and -27 cm (grey) in location 3 of site OSBS and the detected changepoints (coloured triangles) that has at least one match with a changepoint in the time series from another depth.

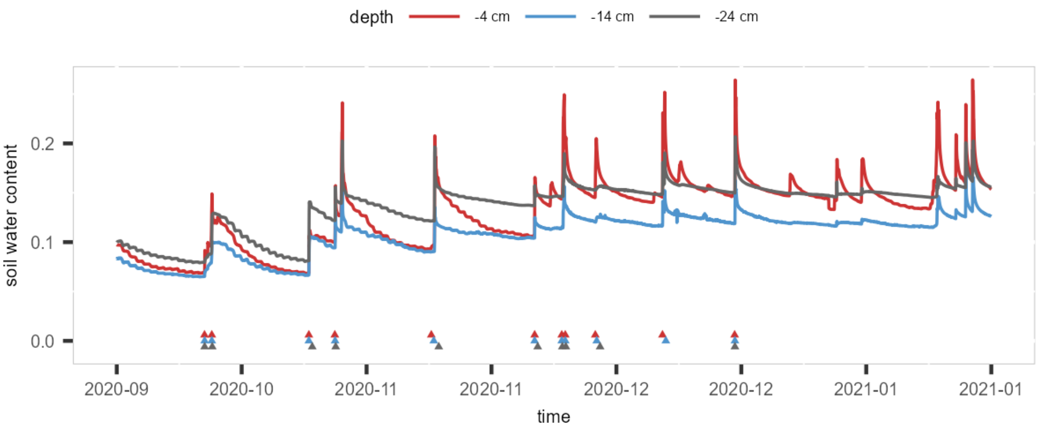

Figure S16: The soil water content time series recorded at -4 cm (red), -14 cm (blue) and -24 cm (grey) in location 2 of site TALL and the detected changepoints (coloured triangles) that has at least one match with a changepoint in the time series from another depth.

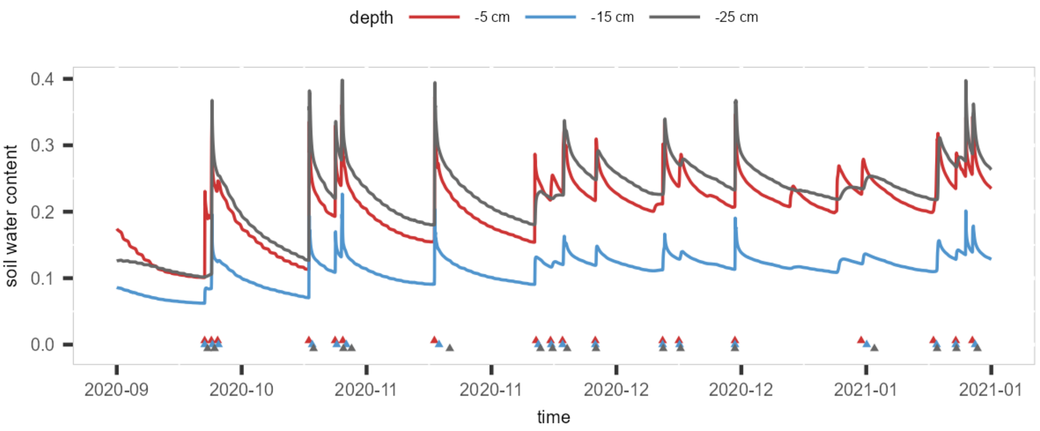

Figure S17: The soil water content time series recorded at -5 cm (red), -15 cm (blue) and -25 cm (grey) in location 3 of site TALL and the detected changepoints (coloured triangles) that has at least one match with a changepoint in the time series from another depth.

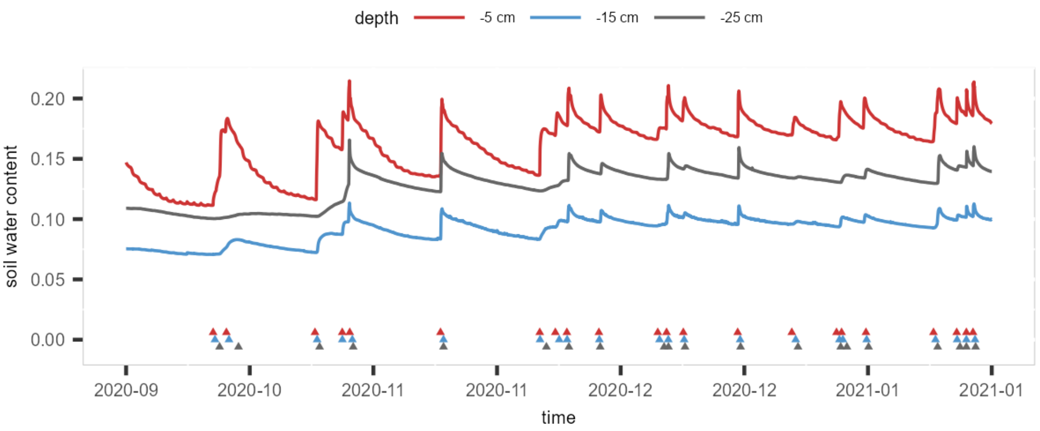

Figure S18: The soil water content time series recorded at -5 cm (red), -15 cm (blue) and -26 cm (grey) in location 4 of site TALL and the detected changepoints (coloured triangles) that has at least one match with a changepoint in the time series from another depth.

References

[1] Gong, M., Killick, R., Nemeth, C., Quinton, J., 2025. A changepoint approach to modelling soil non-stationary moisture dynamics. *Journal of the Royal Statistical Society Series C: Applied Statistics*, 74(3), 866–883. https://doi.org/10.1093/jrsssc/qlaf004

[2] Hastie, T., Tibshirani, R., Friedman, J., 2009. *The elements of statistical learning: data mining, inference, and prediction (2nd Edition)*, New York, Springer

[3] Haynes, K., Eckley, I. A., Fearnhead, P., 2017. Computationally efficient changepoint detection for a range of penalties. *Journal of Computational and Graphical Statistics*, 26(1), 134 -143. https://doi.org/10.1080/10618600.2015.1116445

[4] Hocking, T. D., Rigaill, G., Bach, F., Vert, J., 2013. Learning sparse penalties for change-point detection using max margin interval regression. *Proceedings of 30th International Conference on Machine Learning*, Atlanta, Georgia, USA, 2013. JMLR: W&CP volumn 28.

[5] Killick, R., Fearnhead, P., Eckley, I. A., 2012. Optimal detection of changepoints with a linear computational cost. *Journal of the American Statistical Association*, 107(500), 1590–1598.

[6] Maidstone, R., Hocking, T., Rigaill, G., Fearnhead, P., 2017. On optimal multiple change-point algorithms for large data. *Statistics and Computing*, 27, 519–533.

[7] R Core Team, 2024. R: A language and environment for statistical computing. *R Foundation for Statistical Computing*, Vienna, Austria. https://www.R-project.org/

[8] Nash, J. C., 2016. nlmrt: Functions for Nonlinear Least Squares Solutions. R package version 2016.3.2, https://CRAN.R-project.org/package=nlmrt.

[9] Hengl, T., Mendes de Jesus, J., Heuvelink, G.B., Ruiperez Gonzalez, M., Kilibarda, M., Blagotić, A., Shangguan, W., Wright, M.N., Geng, X., Bauer-Marschallinger, B. and Guevara, M.A., 2017. SoilGrids250m: Global gridded soil information based on machine learning. *PLoS one*, 12(2), p.e0169748

[10] Brouwer, C., Prins, K., Kay, M., and Heibloem, M., 1988. Irrigation Water Management: Irrigation Methods. *Training Manual No. 5*, https://www.fao.org/3/s8684E/s8684e00.htm#Contents

[11] Hillel, D., 2003. Chapter 14: Water enter into soil, in *Introduction to Environmental Soil Physics*, Elsevier Academic Press.

[12] O'Geen, A. T., 2013. Soil Water Dynamics. *Nature Education Knowledge*, 4(5):9. https://www.nature.com/scitable/knowledge/library/soil-water-dynamics-103089121/

[13] Beven, K. J., and Kirkby, M. J., 1979. A physically-based variable contributing area model of basin Hydrology. *Hydrological Sciences Bulletin*, 24, 43–69. https://doi.org/10.1080/02626667909491834
